# Oxidative stress induces Z-DNA-binding protein 1–dependent activation of microglia via mtDNA released from retinal pigment epithelial cells

**DOI:** 10.1016/j.jbc.2021.101523

**Published:** 2021-12-23

**Authors:** Jamal Saada, Ryan J. McAuley, Michela Marcatti, Tony Zifeng Tang, Massoud Motamedi, Bartosz Szczesny

**Affiliations:** 1 Department of Ophthalmology and Visual Sciences, University of Texas Medical Branch, Galveston, Texas, USA; 2 Department of Anesthesiology, University of Texas Medical Branch, Galveston, Texas, USA; 3 Department of Neuroscience, Cell Biology and Anatomy, University of Texas Medical Branch, Galveston, Texas, USA; 4 Department of Neurology, University of Texas Medical Branch, Galveston, Texas, USA

**Keywords:** retinal pigment epithelial, microglia, mitochondrial DNA, extracellular vesicles, oxidative stress, 8-oxoG, 8-hydroxy-2′-deoxy-guanosine, AMD, age-related macular degeneration, BrDU, bromodeoxyuridine, cGAS, cyclic GMP-AMP synthase, CRLBP, cellular retinaldehyde-binding protein 1, EVs, extracellular vesicles, GOx, glucose oxidase, IFN, interferon, IL, interleukins, IP, immunoprecipitation, IRF3, interferon regulatory factor 3, LA-qPCR, long amplicon semiquantitative PCR, LDH, lactate dehydrogenase, MCT3, monocarboxylate transporter 3, mtDNA, mitochondrial DNA, MTT assay, 3-[4,5-dimethylthiazol-2-yl]-2,5 diphenyl tetrazolium bromide assay, NLRP3, nucleotide-binding domain leucine-rich repeat family, pyrin-domain containing 3, NTA, Nanoparticle Tracking Analysis, nuDNA, nuclear DNA, PLA, proximity ligation assay, qPCR, quantitative real-time PCR, RIPK1 and RIPK3, kinases receptor-interacting protein 1 and 3, ROS, reactive oxygen species, RPE, retinal pigment epithelial, STING, stimulator of interferon genes, TBK1, TANK-binding kinase 1, TFAM, mitochondrial transcription factor A, TLR9, Toll like receptor 9, ZBP1, Z-DNA binding protein 1

## Abstract

Oxidative stress, inflammation, and aberrant activation of microglia in the retina are commonly observed in ocular pathologies. In glaucoma or age-related macular degeneration, the chronic activation of microglia affects retinal ganglion cells and photoreceptors, respectively, contributing to gradual vision loss. However, the molecular mechanisms that cause activation of microglia in the retina are not fully understood. Here we show that exposure of retinal pigment epithelial (RPE) cells to chronic low-level oxidative stress induces mitochondrial DNA (mtDNA)-specific damage, and the subsequent translocation of damaged mtDNA to the cytoplasm results in the binding and activation of intracellular DNA receptor Z-DNA-binding protein 1 (ZBP1). Activation of the mtDNA/ZBP1 pathway triggers the expression of proinflammatory markers in RPE cells. In addition, we show that the enhanced release of extracellular vesicles (EVs) containing fragments of mtDNA derived from the apical site of RPE cells induces a proinflammatory phenotype of microglia *via* activation of ZBP1 signaling. Collectively, our report establishes oxidatively damaged mtDNA as an important signaling molecule with ZBP1 as its intracellular receptor in the development of an inflammatory response in the retina. We propose that this novel mtDNA-mediated autocrine and paracrine mechanism for triggering and maintaining inflammation in the retina may play an important role in ocular pathologies. Therefore, the molecular mechanisms identified in this report are potentially suitable therapeutic targets to ameliorate development of ocular pathologies.

Normal retina function is facilitated by an orchestrated interplay between several cell types. The retinal pigment epithelium (RPE) is a highly specialized monolayer of cells with pigmented microvilli, lining the Bruch's membrane located between the neural retina and the choroid in the eye. RPE cells perform several critical functions to maintain retinal homeostasis, including phagocytosis of photoreceptor outer segments, recycling vitamin A, and maintaining the blood–retinal barrier (1). Polarized, mature, and terminally differentiated RPE cells are mitotically quiescent under normal physiological conditions. The RPE is also characterized by an apical-basolateral polarity, with the basolateral surface sitting on the Bruch's membrane and the apical microvilli interfacing with the photoreceptors outer segments. The polarized architecture of the RPE together with tight junctions between cells enables the RPE to form a blood–retinal barrier that is critical for transport of nutrients, ions, and water across the RPE and for directional secretion of growth factors and extracellular matrix components to help maintain structure and function of the photoreceptors and choroids (1, 2). Thus, considering the critical role of RPE in maintenance of retinal homeostasis, dysfunctional RPE is observed in several retinopathies. The best studied example of RPE-initiated ocular pathology is age-related macular degeneration (AMD). Many experimental and clinical data support the view that damage to RPE cells by enhanced oxidative stress is a crucial factor in AMD development (3, 4, 5, 6). Morphological changes in the RPE are also frequently involved in hereditary retinopathies such as retinitis pigmentosa where progressive degeneration of rods and cones is directly linked with RPE dysfunction (7).

Damage to mitochondrial DNA (mtDNA) together with mitochondrial dysfunction is well recognized in AMD, but they are currently viewed as a passive downstream effect of oxidative/inflammatory injury (8, 9, 10, 11, 12, 13, 14). It is established that in comparison to nuclear DNA (nuDNA), mtDNA is highly susceptible to oxidative injury in several cell types, including post mitotic cells (15, 16, 17). Also, from an immunological point of view, since several characteristics of mtDNA bear a resemblance to bacterial origin (small size, multiple copies, methylation status), it is recognized by immune cells as “foreign” not “self” DNA when is released to cytoplasm or extracellular space (18). Damaged mtDNA liberated from necrotic cells was shown to induce inflammatory responses, mostly in immune cells, by binding to several distinct RNA/DNA receptors in various diseases (18). However, the importance of these DNA/RNA receptors in ocular pathologies is just starting to emerge.

Activation of microglia, resident immune cells of the central nervous system including the retina, is directly linked with development of various ocular pathologies. Microglia play a critical role in homeostatic maintenance of the neuroretinal microenvironment (19). In the normal, healthy retina, microglia are located in the inner retina, the ganglion cell layer, and inner and outer plexiform layer, where they exhibit branched cellular processes responsible for immune surveillance. Trauma or noxious insults to the retina such as oxidative stress, hypoxia, or inherited mutations trigger microglia reactivity characterized by amoeboid morphology, increased proliferation, and migration to the site of injury (20). While the initial “beneficial” inflammatory response can rapidly enhance tissue repair and a return to homeostasis, chronic activation of microglia induces severe alterations in retinal integrity and aggravates neuronal death (19, 20). It has been proposed that pathological microglia and RPE cell cross talk in the subretinal space results in alterations to the structure and physiology of the RPE layer, which in turn transforms the retinochoroidal interface into a favorable environment for the progression and advancement of several ocular pathologies, including AMD (21). Activated microglia induce RPE alterations that result in an elevated chemoattractant, proinflammatory, and proangiogenic environment, which increases the recruitment and activation of immune cells and fosters the growth of neovascular vessels into the retina. In hereditary retinopathies such as retinitis pigmentosa, chronically activated microglia are engaged in the phagocytosis of rod debris and also exacerbate photoreceptors and other adjacent cells by secretion of proinflammatory neurotoxic factors. The critical roles of microglia in neurodegeneration of retinal ganglion cells in glaucoma and diabetic retinopathies have also been recognized (19, 21). Although substantial advances have recently been made in understanding mechanisms of microglia activation, the communication between microglia and other retina-specific cell types is still not well defined.

Oxidative stress, RPE dysfunction, and activation of the proinflammatory phenotype of microglia are commonly observed in ocular pathologies. In this project, we investigated the effect of chronic oxidative stress on communication between RPE cells and microglia. Our study revealed a unique RPE–microglia cross talk mediated by extracellular vesicles (EVs) and illuminated the importance of autocrine and paracrine signaling mediated by oxidatively damaged mtDNA. Moreover, we identified a pathological role of damaged mtDNA through its interaction with the immune DNA receptor, Z-DNA-binding protein 1 (ZBP1), in the development of a pathological proinflammatory environment in the retina, which may serve as a novel therapeutic target for ocular pathologies.

## Results

### Detrimental effect of chronic low-level oxidative stress on metabolic status, cellular viability, and DNA damage of differentiated ARPE-19 cells

**Figure 1:**
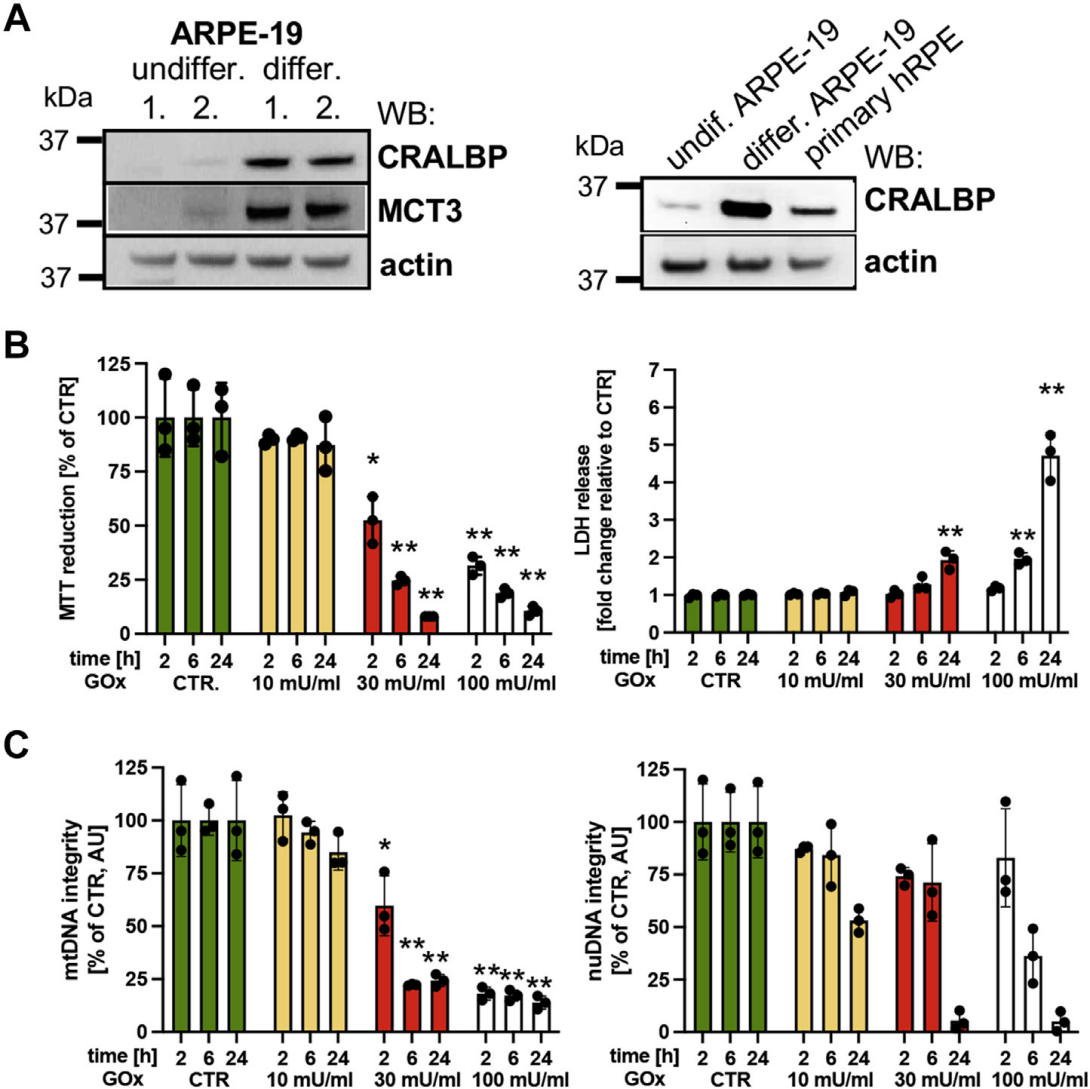
**Differentiated ARPE-19 cells are highly sensitive to chronic low-level oxidative stress.***A,* Western blot analysis of cellular retinaldehyde-binding protein 1 (CRLBP) and monocarboxylate transporter 3 (MCT3) in undifferentiated (replicating), differentiated (nonreplicating cells maintained for 3 months in medium containing 1% FBS) ARPE-19 cells and primary human RPE cells. Representative images of n = 3 are shown. *B,* time-dependent changes of cellular viability measured by the metabolic status (*left panels*) and necrotic cell death (*right panels*) of ARPE-19 cells in response to the increasing concentration of glucose oxidase (GOx) treatment. *C,* time-dependent changes in mtDNA (*left panels*) and nuDNA (*right panels*) integrity in ARPE-19 cells in response to the increasing concentration of GOx treatment. Data are based on n = 3 and are expressed as mean ± SD. ∗*p* < 0.05, ∗∗*p* < 0.01 *vs.* control (untreated) cells based on one-way ANOVA multiple comparison tests.

In this study, we used differentiated human retinal pigment epithelial cells (ARPE-19) that have been shown to regain characteristics of primary RPE cells after long-term culturing under low-serum conditions (22). ARPE-19 cells were maintained for at least 3 months in medium containing 1% FBS and their differentiation was confirmed by Western blot analysis of cellular retinaldehyde-binding protein 1 (CRLBP), a protein known to be associated with the vision cycle and expressed only in fully differentiated RPE cells (22). We also tested the protein level of monocarboxylate transporter 3 (MCT3) that was shown to be expressed only in fully differentiated and polarized ARPE-19 cells (23). In agreement with these reports, unlike undifferentiated, proliferating ARPE-19 cells, the differentiated ARPE-19 and primary human RPE cells showed marked expression of both CRLBP and MCT3 (Figs. 1*A* and S1). In subsequent experiments, we used only fully differentiated ARPE-19 cells.

Virtually all ocular pathologies that progress over time are linked with chronic, low-level oxidative stress as a major contributing factor for the development of pathological characteristics. Although most studies to date utilized H_2_O_2_ directly to induce oxidative stress, H_2_O_2_ treatment induces bolus oxidative stress that is not physiologically relevant, particularly for studies of time-dependent pathologies. Therefore, to mimic chronic oxidative stress, we used a glucose oxidase (GOx) treatment that can enzymatically generate constant low levels of H_2_O_2_ over an extended period of time (24). The amount of H_2_O_2_ generated by GOx was measured with Amplex Red and compared with a standard curve generated with H_2_O_2_ (Fig. S2*A*). We measured that 10 mU/ml of GOx generated 40 μM, 60 μM, and 100 μM of H_2_O_2_ within 1, 3, and 6 h, respectively (Fig. S2*B*). Next, we compared the effects of GOx and direct H_2_O_2_ exposure by analysis of metabolic status and necrosis in ARPE-19 cells, using MTT assay and release of lactate dehydrogenase (LDH), respectively, and detected a marked difference. As an example, 30 mU/ml of GOx (which generates 300 μM of H_2_O_2_ within 6 h) reduced MTT by 50%, 80% and 95% at 2, 6, and 24 h, respectively, while a modest (less than 20%) decrease of MTT was measured only at 2 h post 300 μM H_2_O_2_ treatment (Figs. 1*B* and S3*A*). Similarly, treatment with 100 mU/ml of GOx (which generates 1 mM H_2_O_2_ within 6 h) induced a two- and fivefold increase of LDH release at 6 h and 24 h, while a twofold increase in LDH release was measured only at 2 h post 1 mM H_2_O_2_ treatment (Figs. 1*B* and S3*A*). In addition, we tested the effects of GOx and H_2_O_2_ treatments on DNA damage measured by a long amplicon semiquantitative PCR (LA-qPCR) method that allows for concurrent measurement of nuclear and mitochondrial DNA (nuDNA and mtDNA) damage (25). Similarly, as above, the dynamics of oxidatively induced DNA damage and its repair in differentiated ARPE-19 cells treated with GOx and H_2_O_2_ is different. The 30 mU/ml of GOx (equivalent to 300 μM H_2_O_2_) induced a 40% and 80–90% decrease of mtDNA integrity at 2, 6, and 24 h, respectively, together with a significant decrease of nuDNA integrity measured at 24 h (Fig. 1*C*). In comparison, direct treatment of ARPE-19 cells with 300 μM of H_2_O_2_ has no effect on mtDNA and nuDNA integrity at any time points (Fig. S3*B*). Similarly, treatment of ARPE-19 cells with 100 mU/ml of GOx (equivalent to 1 mM H_2_O_2_) induced an 80% decrease of mtDNA as early as 2 h, with a 60% decrease in integrity of nuDNA as early as 6 h (Fig. 1*C*). However, direct treatment with 1 mM H_2_O_2_ induced reduction of mtDNA integrity by 25% only at 2 h, which is subsequently repaired by 6 h. At this concentration of direct H_2_O_2,_ no measurable changes in the integrity of nuDNA were observed (Fig. S3*B*). Together, these data show a marked different cellular response to bolus H_2_O_2_ treatment and chronic oxidative stress induced by GOx. We detected detrimental effects of chronic, low-level oxidative stress at a relatively low concentration of H_2_O_2_. We calculated that at least one order of magnitude less H_2_O_2_ generated by GOx has more significant effects on cellular viability as compared with direct H_2_O_2_ treatment. This observation is particularly important in studies of age or time-dependent pathologies where the effects of prolonged exposure to low levels of oxidants are suspected to drive pathological changes. In subsequent experiments, we use a sublethal concentration of GOx (30 mU/ml) to investigate its effects in differentiated ARPE-19 cells.

### Oxidatively damaged mtDNA binds to the cytoplasmic DNA receptor, Z-DNA binding protein 1

**Figure 2:**
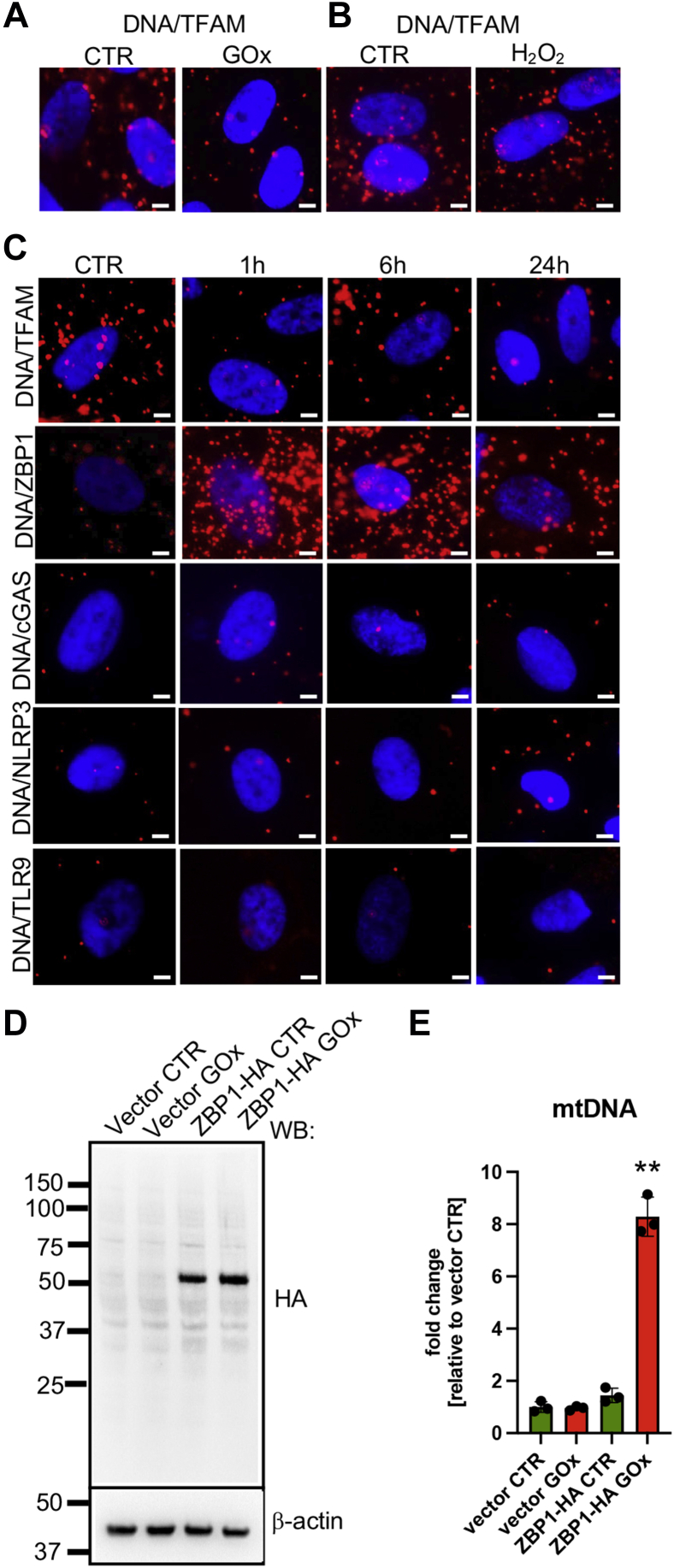
**Chronic oxidative stress causes translocation of mtDNA to the cytoplasm to bind to the ZBP1 receptor in differentiated ARPE-19 cells.** Analysis of binary interaction between mtDNA and TFAM in response to (*A*) 30 mU/ml of GOx and (*B*) 300 μM H_2_O_2_ visualized with PLA at 1 h post treatment. Note the marked decrease DNA/TFAM interaction in cells treated with GOx, but not with H_2_O_2_. *C,* time-dependent changes in interactions between mtDNA and intracellular DNA receptors (ZBP1, Z-DNA-binding protein 1; cGAS, cyclic GMP-AMP synthase; NLRP3, NOD, LRR and pyrin domain-containing protein 3 inflammasome; TLR9, toll-like receptor 9) in cells treated with 30 mU/ml of GOx. Please note the early stable interaction occurs only between mtDNA and ZBP1, and there is extranuclear localization of the signal. Representative images of n = 3 are shown. Scale bar, 10 μm. *D,* Western blot analysis of ZBP1-HA expression at 48 h post transfection with control or pCAGGS-HA-hZBP1 in ARPE-19 cells. *E,* the amounts of mtDNA and nuDNA in ZBP1s′ IPs in control and GOx-treated ARPE-19 cells. Data are based on n = 3 and are expressed as mean ± SD. ∗∗*p* < 0.01 *vs.* vector control based on one-way ANOVA multiple comparison tests.

We previously reported that exposure of replicating lung epithelial cells to oxidative stress causes mtDNA-specific damage and its translocation to the cytoplasm where it then binds and activates ZBP1, inducing the expression of proinflammatory markers (16). Here, we confirmed that differentiated ARPE-19 cells are also highly susceptible to mtDNA damage from oxidative injury (Fig. 1*C*). Since chronic inflammation is commonly observed in many pathologies including age-related macular degeneration, glaucoma, diabetic retinopathies, and others, we tested the effects of chronic oxidative stress on intracellular translocation of mtDNA using proximity ligation assay (PLA). PLA detects the binary interaction between two epitopes *in cellulo* based on antibody specificity (26). First, we tested the interaction between mtDNA and mitochondrial transcription factor A (TFAM), which is known to stably interact with mtDNA in mitochondria under physiological conditions (27). We compared interactions between mtDNA/TFAM in response to 30 mU/ml of GOx and 300 μM H_2_O_2_ treatment. While a marked reduction of the mtDNA/TFAM signal at 1 h was detected in ARPE-19 cells treated with GOx (Fig. 2*A*), no effect on mtDNA/TFAM interactions post direct H_2_O_2_ treatment was observed (Fig. 2*B*). These results further confirm different cellular outcomes to exposure of chronic and bolus oxidative stress. We hypothesized that GOx-induced mtDNA damage (Fig. 1*C*) caused the dissociation between mtDNA and TFAM (Fig. 2*A*) resulting in translocation of damaged mtDNA to the cytoplasm. Therefore, at various time points, we tested GOx treatment on interaction between mtDNA and several intracellular DNA/RNA receptors (18). Using PLA we detected marked increased interaction between mtDNA and ZBP1 as early as 1 h, which was maintained at 6 h and 24 h post GOx treatment (Fig. 2*C*). We did not detect interaction between mtDNA and Toll-like receptor 9 (TLR9), cyclic GMP-AMP synthase (cGAS), or nucleotide-binding domain leucine-rich repeat family, pyrin-domain containing 3 (NLRP3) at any time points post GOx treatment (Fig. 2*C*). Since PLA depends highly on antibody specificity, we further confirmed intracellular localization of PLA signal (mtDNA/TFAM and mtDNA/ZBP1) by costaining PLA slides with the mitochondrial marker, subunit B of ATP synthase (ATPB). As expected, we observed colocalization of mtDNA/TFAM PLA signal with mitochondria in control but not GOx-treated cells and lack of colocalization of mtDNA/ZBP1 PLA signal in mitochondria of GOx-treated cells (Fig. S4). Since PLA depends on antibodies specificity, we independently confirm the interaction between mtDNA and ZBP1 in response to prolonged oxidative stress, by expressing HA-tagged ZBP1 in differentiated ARPE-19 cells (Fig. 2*D*) and tested for the presence of mtDNA and nuDNA in ZBP1-HA immunoprecipitated samples (IPs). More than eightfold increase of the mtDNA in ZBP1 IPs was measured with qPCR under GOx treatment, but not in control cells (Fig. 2*E*) with negligible amounts of nuDNA detected in IPs (Fig. S5). Together, these data showed that under chronic oxidative stress, oxidatively damaged mtDNA is translocated to the cytoplasm to stably bind cytoplasmic receptor ZBP1.

### Inflammatory response triggered by activation of ZBP1 signaling by mtDNA

**Figure 3:**
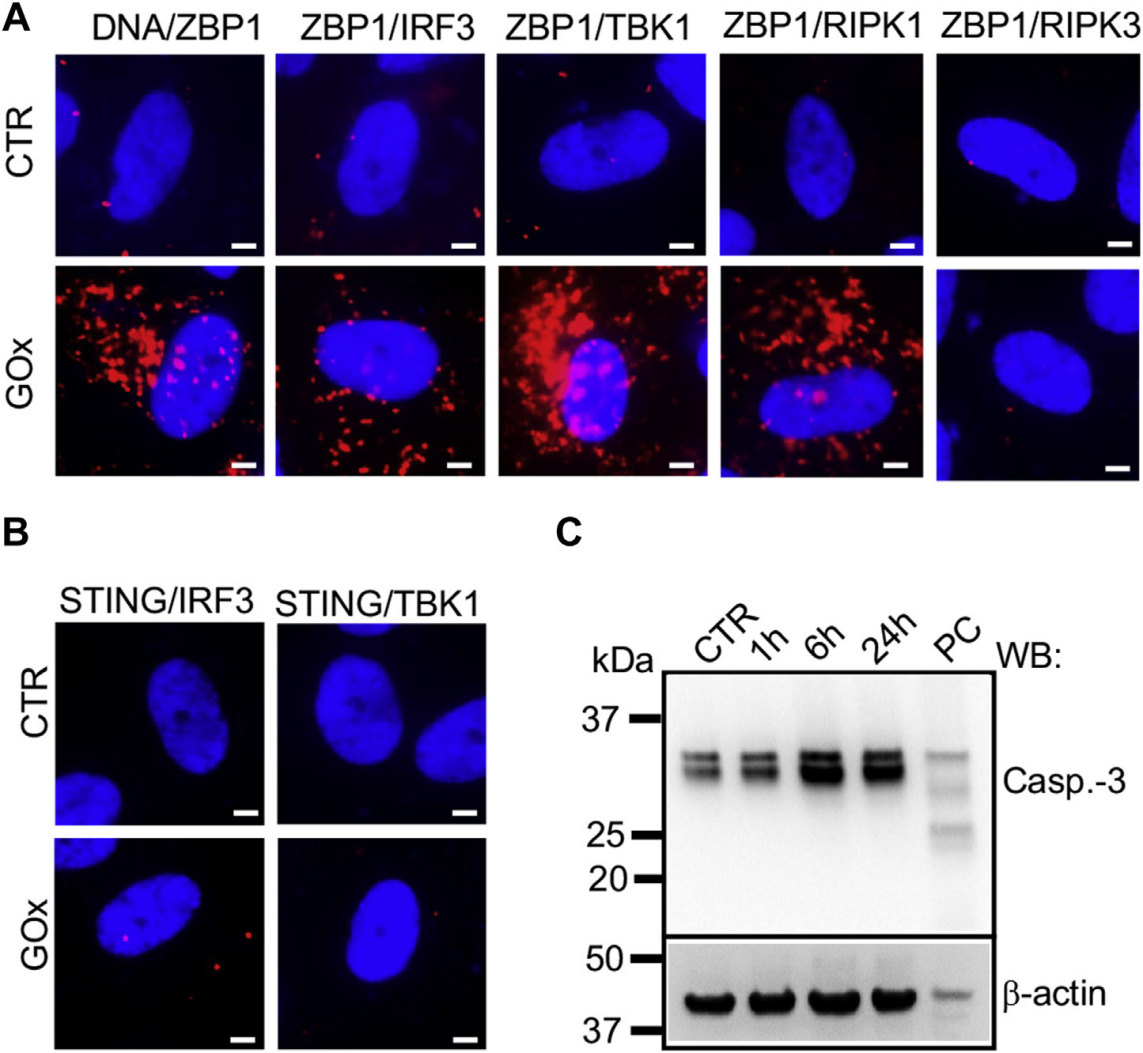
**Chronic oxidative stress activates ZBP1/RIPK1-mediated inflammatory response in differentiated ARPE-19 cells.***A,* activation of ZBP1/RIPK1 signaling pathway was analyzed using PLA. Interaction between mtDNA/ZBP1, ZBP1/IRF3, ZBP1/TBK1, ZBP1/RIPK1 but not ZBP1/RIPK3 was detected at 1 h post 30 mU/ml of GOx treatment using a PLA. *B,* 30 mU/ml of GOx does not activate cGAS/STING pathway as determined by the lack of interaction between STING/IRF3 and STING/TBK1 by PLA. Representative images of n = 3 are shown. Scale bar, 10 μm. *C,* lack of caspase-3 activation in ARPE-19 cells analyzed by Western blotting at several time points post 30 mU/ml of GOx treatment. PC, positive control (100 mU/ml of GOx).

**Figure 4:**
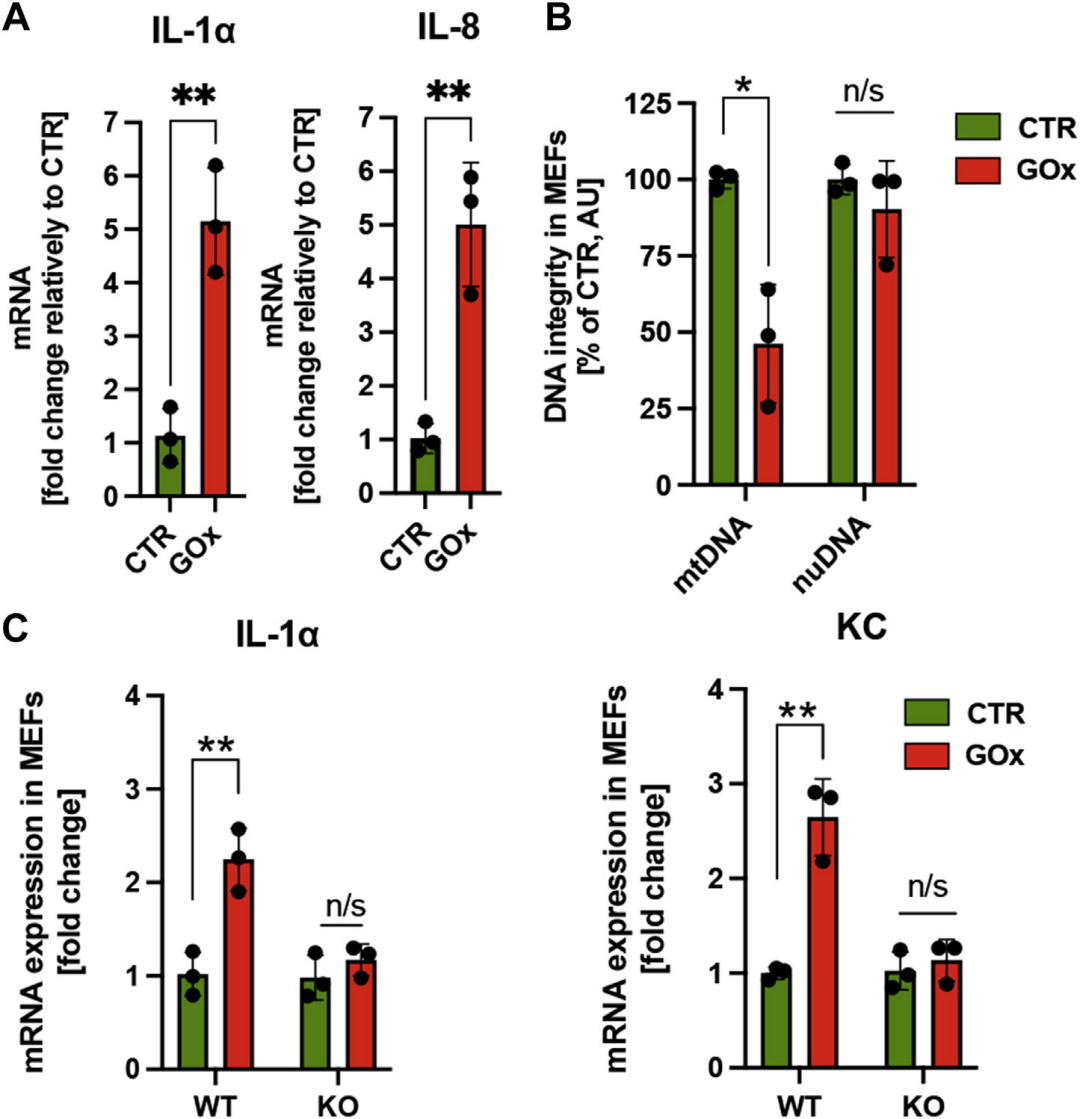
**Chronic oxidative stress induces expression of proinflammatory markers via activation of ZBP1 signaling.***A,* enhanced expression of IL-1α and IL-8 in differentiated ARPE-19 cells at 24 h posttreatment with 30 mU/ml of GOx. *B,* mtDNA-specific damage in mouse embryonic fibroblast (MEFs) derived from wild-type (WT) C56BL/6J animals subjected to 30 mU/ml of GOx. *C,* increased expression of proinflammatory markers (IL-1α and KC, mouse equivalent of human IL-8) in the WT but not in the ZBP1 KO MEFs subjected to 30 mU/ml of GOx at 24 h. Please note, ZBP1 depletion inhibits the expression of proinflammatory markers induced by mtDNA-specific damage. Data are based on n = 3 and are expressed as mean ± SD. ∗*p* < 0.05; ∗∗*p* < 0.01 *versus* control (untreated with GOx) cells based on *t*-tests.

The role of ZBP1 is recognized in virally induced inflammatory responses and programmed cell death (PANoptosis), including regulated pyroptosis (programmed inflammatory cell death), necroptosis (programmed necrosis), and apoptosis (28). To investigate which ZBP1-mediated signaling pathway is activated by oxidatively damaged mtDNA (Fig. S6), we tested the interaction of ZBP1 with interferon regulatory factor 3 (IRF3), TANK-binding kinase 1 (TBK1), and kinases receptor-interacting protein 1 and 3 (RIPK1 and RIPK3) in differentiated ARPE-19 cells treated with 30 mU/ml of GOx for 2 h. We detected interactions between ZBP1 and RIPK1, IRF3, TBK1, but not with RIPK3, indicating activation of the noninflammasome-mediated inflammatory response (Fig. 3*A*). Because activation of the cGAS/stimulator of interferon genes (STING) pathway was recently suggested in AMD (29), we tested the effects of GOx treatment on the interaction between STING and IRF3 or TBK1. The lack of interaction between these proteins rules out the activation of cGAS/STING pathway induced by chronic oxidative stress in ARPE-19 cells (Fig. 3*B*), which further supports a lack of interaction between mtDNA and cGAS (Fig. 2*C*). Moreover, the lack of activation of ZBP1-mediated cell death in GOx-treated ARPE-19 cells is supported by the absence of significant necrotic cell death, as measured by the LDH released by 30 mU/ml of GOx treatment (Fig. 1*B*) and lack of activation of caspase-3 (Fig. 3*C*). To test our conclusion that prolonged exposure to low oxidative stress results in inflammation, we measured the expression of several proinflammatory markers in response to GOx treatment at 24 h and detected a robust increase of IL-1α and IL-8 mRNA levels (Figs. 4*A* and S7). To confirm the key role of ZBP1 in the expression of proinflammatory markers, we used mouse embryonic fibroblasts (MEFs) derived from wild-type (WT) and ZBP1 KO mice (Fig. S8). As observed with ARPE-19 cells, significant mtDNA-specific damage was measured in MEFs subjected to 30 mU/ml of GOx at 1 h (Fig. 4*B*). Most importantly, a significant reduction of IL-1α, and KC (mouse equivalent of human IL-8) expression was measured in GOx-treated MEFs derived from ZBP1 KO mice in comparison to WT (Fig. 4*C*). These data strongly support the key role of mtDNA-mediated activation of ZBP1 signaling in generating a proinflammatory environment in response to prolonged oxidative stress in differentiated RPE cells.

### Chronic oxidative stress induces release of extracellular vesicles (EVs) containing large fragments of mtDNA from differentiated ARPE-19 cells

**Figure 5:**
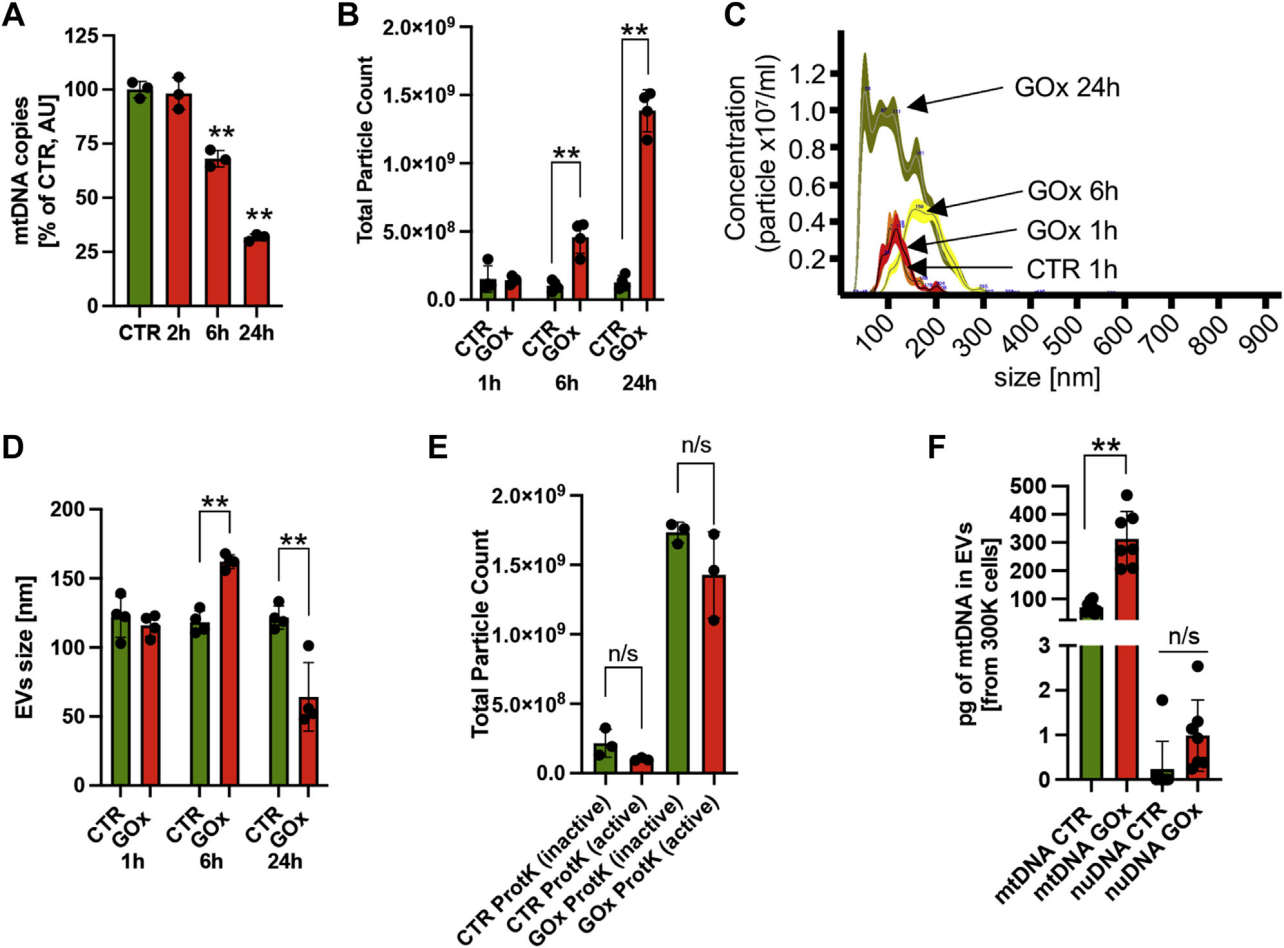
**Chronic oxidative stress causes release of EVs containing mtDNA from differentiated ARPE-19 cells.** Time-dependent changes in (*A*) mtDNA content in GOx-treated ARPE-19 cells measured by qPCR, (*B*) total number of particles (EVs) in culture medium of GOx-treated ARPE-19 cells, and (*C*) particle size profiles determined by NTA analysis derived from 300,000 cells. *D,* GOx-induced first release of larger (at 6 h) and later smaller (at 24 h) EVs. *E,* lack of effect of Proteinase K treatment on the total number of particles released from ARPE-19 cells indicates the GOx treatment particle increase is not due to protein aggregates (media containing EVs at 24 h CTR and GOx samples were analyzed). *F,* increased amounts of mtDNA in EVs isolated at 24 h post GOx-treatment of ARPE-19 cells. Data are based on n = 3 and are expressed as mean ± SD. ∗∗*p* < 0.01 *vs.* control (untreated with GOx) cells based on one-way ANOVA multiple comparison tests (*A*) or *t*-tests (*B*, *D*, and *F*).

**Figure 6:**
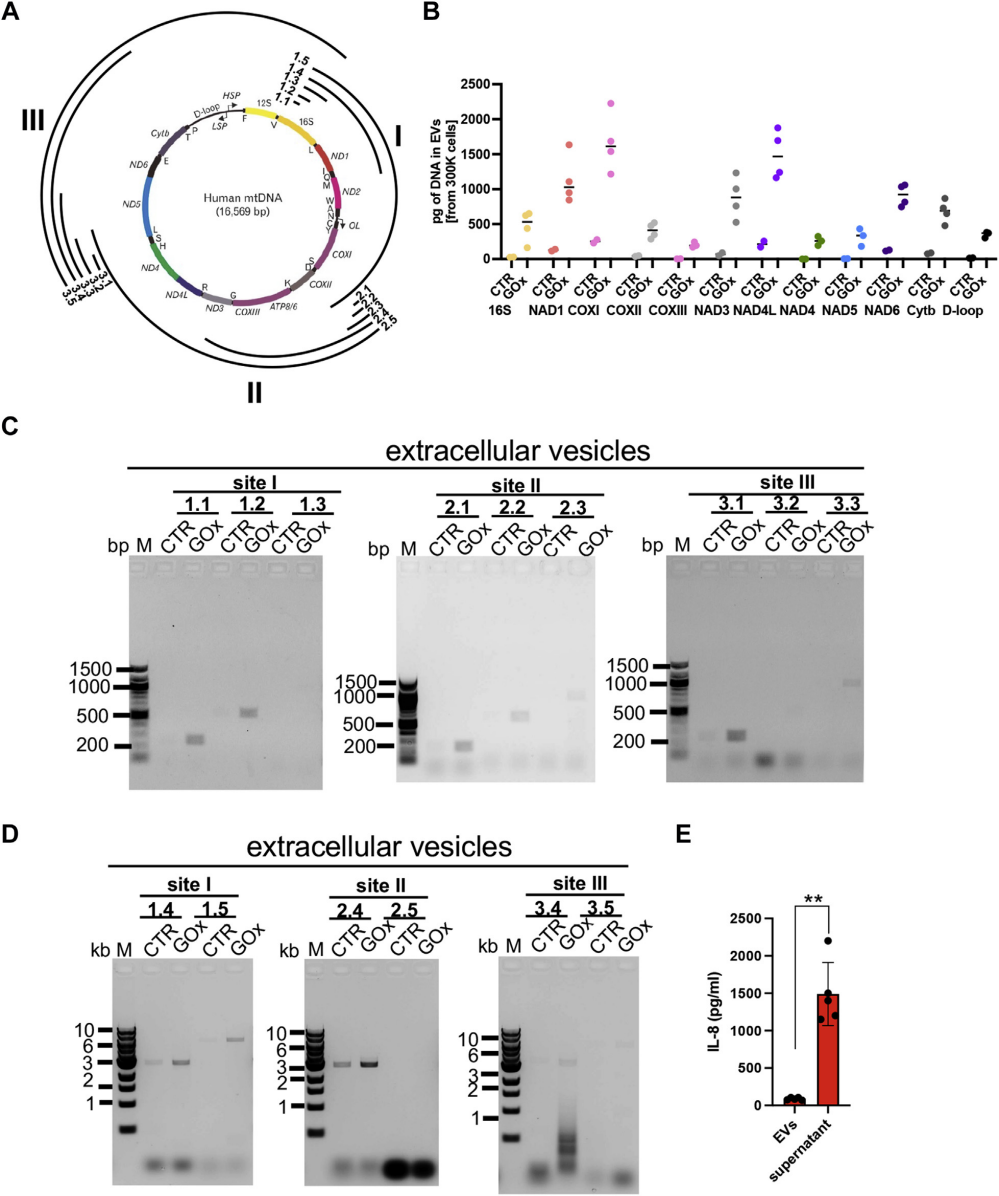
**EVs released from differentiated ARPE-19 cells contain large fragments of mtDNA.***A,* schematic representation of human mtDNA together with primers used in analysis. *B,* all tested genes encoded by mtDNA are present in EVs released from GOx-treated ARPE-19 cells as measured by qPCR. Analysis of the mtDNA fragment size in EVs released at 24 h from the same number of CTR and GOx-treated ARPE-19 cells was performed. The amplicons of (*C*) 200 bp–1 kb and (*D*) 3–6 kb were analyzed. *E,* comparison of IL-8 in isolated EVs and supernatant (medium) of ARPE-19 cells treated with 30 mU/ml of GOx. EVs were isolated at 24 h post GOx-treatment. Representative images on n = 3 are shown (*C* and *D*). Please note enhanced amounts of amplicons in EVs isolated particularly from GOx-treated ARPE-19 cells. Graph based on n = 5 is expressed as mean ± SD. ∗∗*p* < 0.01 IL-8 in EVs *vs.* supernatant based on *t*-tests (*E*).

We tested whether susceptibility of mtDNA to prolonged oxidative stress (Fig. 1*C*) has effects on mitochondrial respiration. All major bioenergetic parameters, namely oxygen consumption linked with basal respiration, maximal respiration, and ATP production, were significantly reduced at 3 h and 24 h post GOx treatment, with a marked increase in proton leakage (Fig. S9). Interestingly, we also detected a time-dependent decrease of mtDNA content in ARPE-19 cells treated with 30 mU/ml of GOx (Fig. 5*A*), suggesting that oxidatively damaged mtDNA is translocated to cytoplasm to bind ZBP1, but is also subjected to degradation or extrusion to the extracellular space. To test the latter, we measured the number of particles derived from control and GOx-treated differentiated ARPE-19 cells using Nanoparticle Tracking Analysis (NTA) and detected an almost tenfold increase in total particle count (Fig. 5*B*). Interestingly, time-dependent analysis of the particle sizes revealed that larger particles (∼150 nm) are released earlier and smaller (∼60 nm) are released at later time points following GOx treatment (Fig. 5, *C* and *D*). Importantly, 90% of released EVs are smaller than 200 nm, ruling out the presence of apoptotic bodies in the collected media of GOx-treated cells and independently confirming a lack of apoptosis in our experimental conditions (Fig. 5*C*). We also detected the same profile of EVs in precleared (three subsequent low-speed centrifugations) medium and post ultracentrifugation (Fig. S10*A*) and showed that almost all EVs present in culture medium can be sedimented by ultracentrifugation (Fig. S10*B*). To test whether protein aggregates are generated by GOx treatment as opposed to EVs, we treated precleared media with either active or denatured (inactive) Proteinase K. We found no significant decrease in total particle count under Proteinase K treatment, which serves as confirmation that the particles present are EVs released from differentiated ARPE-19 cells (Fig. 5*E*). Next, we compared amounts of mtDNA and nuDNA in EVs sedimented by ultracentrifugation and detected a threefold increase of mtDNA in EVs derived from GOx-treated cells as compared with control and only negligible amounts of nuDNA in both treated and control samples (Fig. 5*F*). The majority of mtDNA is present in sedimented EVs but not in the remaining medium (Fig. S10*C*). Then, we investigated the properties of the mtDNA by analyzing sequence specificity and fragment size of mtDNA present in EVs. Using qPCR, we detected increased amounts of the most of mtDNA-encoded genes (Fig. 6*A*) in EVs derived from GOx-treated ARPE-19 cells, in comparison to the control (Fig. 6*B*). This further confirmed enhanced amounts of mtDNA in EVs derived from GOx-treated cells. To investigate fragment sizes of the mtDNA in EVs, we designed primers to amplify mtDNA fragments of 200 bp up to 6 kb, localized in three different regions of the mtDNA genome (Figs. 6*A* and S11). We were able to amplify the majority of the mtDNA fragments, particularly in EVs derived from GOx-treated cells (Fig. 6, *C* and *D*). In addition, we also determined that IL-8, which exhibits increased expression in ARPE-19 cells treated with GOx (Fig. 4*A*), is released to the culture medium but not within EVs (Fig. 6*E*). Taken together, we concluded that low-level oxidative stress enhances the release of EVs and increase amounts of mtDNA fragments of various sizes (up to 6 kb) in EVs derived from differentiated ARPE-19 cells.

### EVs containing mtDNA derived from oxidatively stressed, polarized ARPE-19 cells activate microglia

**Figure 7:**
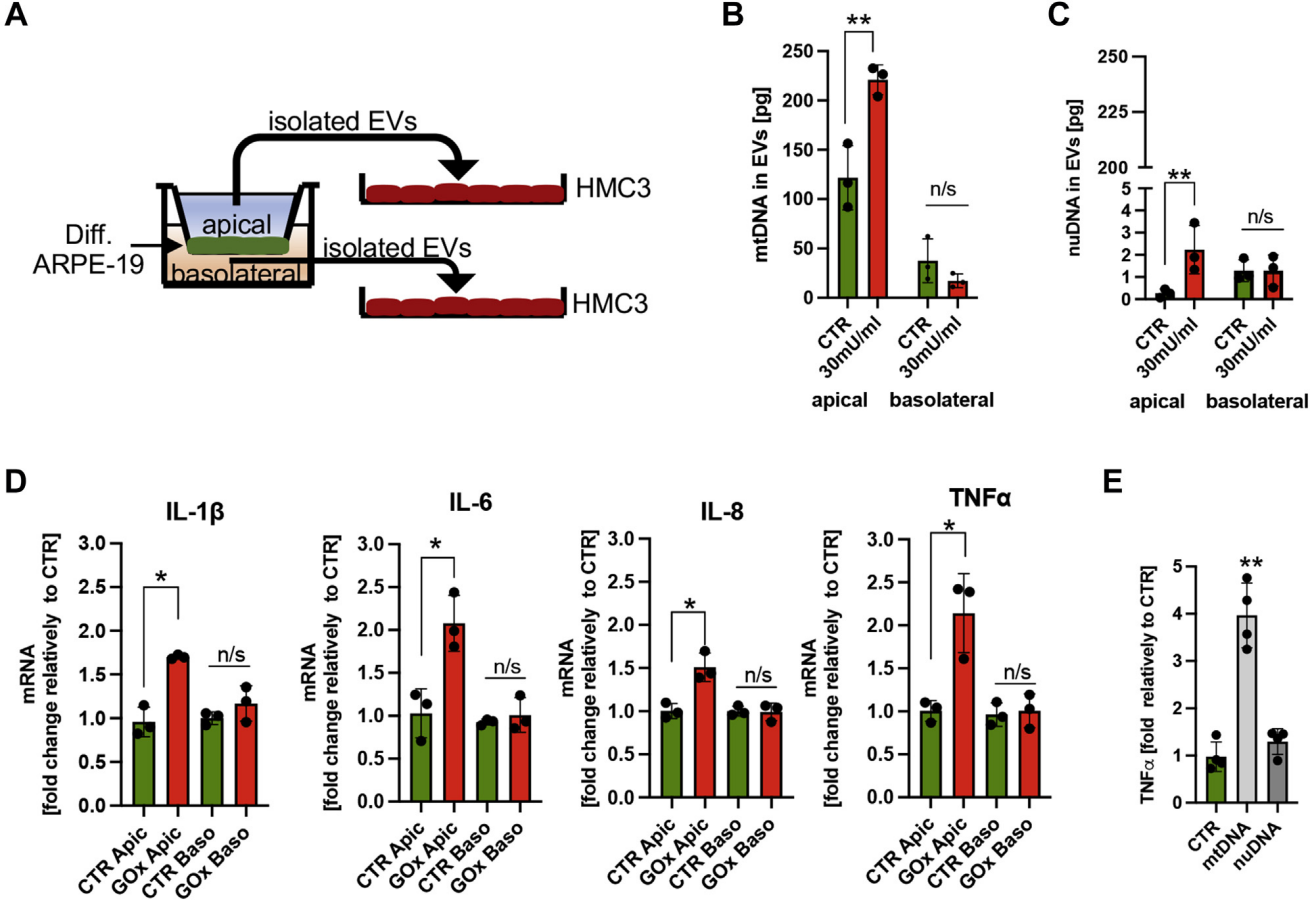
**EVs containing mtDNA derived from apical sites of differentiated ARPE-19 cells activate microglia.***A,* schematic representation of the experiment using ARPE-19 cells differentiated in a transwell plate. Quantification of (*B*) mtDNA and (*C*) nuDNA in EVs isolated from apical and basolateral sites of control and ARPE-19 cells treated with 30 mU/ml of GOx at 24 h posttreatment. Note, an increased amount of mtDNA in EVs released from the apical site of ARPE-19 cells post GOx treatment. 500-fold more mtDNA than nuDNA was measured in EVs isolated from the apical site of GOx-treated cells. *D,* enhanced expression of proinflammatory cytokines in microglia treated with EVs isolated from the apical site of GOx-treated ARPE-19 cells. *E,* robust expression of TNFα in microglia treated with isolated mtDNA. Data are based on n = 3 and are expressed as mean ± SD. ∗*p* < 0.05, ∗∗*p* < 0.01 *vs.* control/apical site based on *t*-tests (for *B*, *C*, and *D*) and one-way ANOVA multiple comparison tests (*E*).

Fully differentiated ARPE-19 cells are characterized by an apical-basolateral polarity (2). Therefore, we tested the amount of DNA in EVs released from the apical and basolateral sites of differentiated ARPE-19 cells isolated at 24 h post GOx treatment (Fig. 7*A*). We measured two orders of magnitude more mtDNA than nuDNA in EVs released from control and GOx-treated cells (Fig. 7, *B* and *C*). We also measured markedly more mtDNA released from the apical site than from the basolateral site, which was further enhanced (twofold increase) in cells subjected to GOx (Fig. 7*B*). More nuDNA was found in EVs released from the basolateral site than from the apical site, but its amount was reduced in GOx-treated ARPE-19 cells (Fig. 7*C*). To test the importance of ARPE-19 cell polarity on EVs release, we also measured the amount of nuDNA and mtDNA in EVs released from apical and basolateral sites of undifferentiated, replicating ARPE-19 cells. Generally, as with polarized, differentiated ARPE-19 cells, more mtDNA than nuDNA was measured in EVs of undifferentiated cells (Fig. S12). However, opposite to polarized ARPE-19 cells, EVs isolated from the basolateral site of replicating ARPE-19 cells contained more mtDNA and its amount did not change post GOx treatment (Fig. S12*A*). Also, in contrast to differentiated ARPE-19 cells, more nuDNA was found in EVs released from the apical site, which decreased post GOx treatment (Fig. S12*B*). These data emphasize the importance of testing fully differentiated and polarized ARPE-19 cells that closely mimic the primary RPE cells. Together, we found a marked difference in the amount of mtDNA in EVs released from the apical and the basolateral site suggesting the possibility of different roles for EVs released from polarized differentiated ARPE-19 cells.

**Figure 8:**
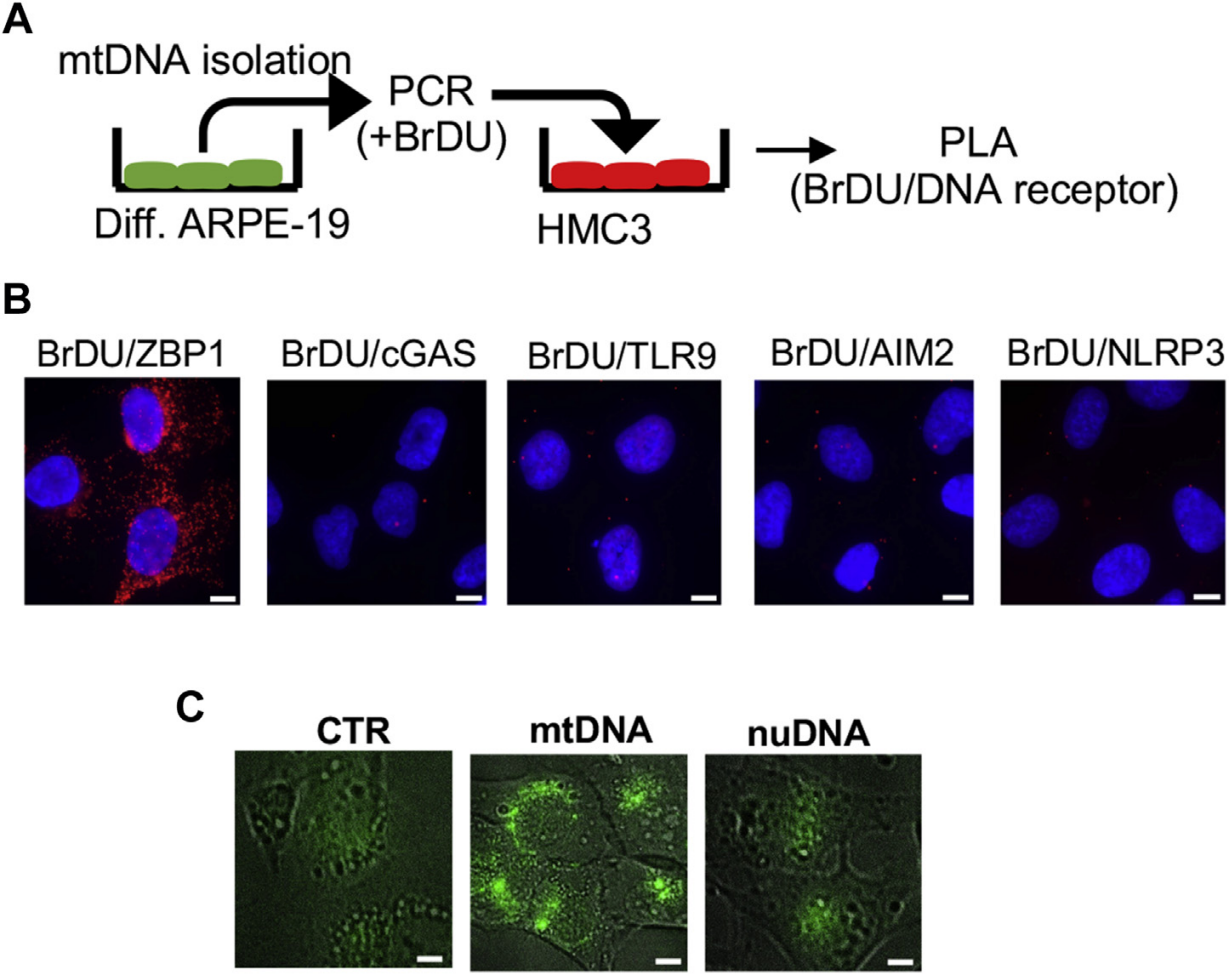
**Microglial ZBP1 binds mtDNA derived from differentiated ARPE-19 cells.***A,* schematic representation of the experiment. *B,* PLA analysis between BrDU-labeled mtDNA isolated from ARPE-19 cells and intracellular receptors in microglia. Please note that only ZBP1 binds mtDNA. *C,* enhanced production of reactive oxygen species in microglia treated with isolated mtDNA as indicated by MitoSOX *green staining*. Representative images of n = 3 are shown. Scale bar, 10 μm.

Since microglia activation has been linked to many ocular pathologies (19, 20), we tested the effects of EVs released from RPE cells on microglia activation. ARPE-19 cells differentiated on transwell plates were subjected to 30 mU/ml GOx for 24 h, and EVs isolated from apical and basolateral sites were used to treat unstimulated HMC3 cells (human microglia), followed by measurement of the expression level of several cytokines (Fig. 7*A*). We detected a significant increase in the mRNA of proinflammatory markers (IL-1β, IL-6, IL-8, and TNFα) at 24 h in HMC3 treated with EVs isolated from the apical site of GOx-treated, but not in control ARPE-19 cells (Fig. 7*D*). In addition, EVs isolated from the basolateral site of control or GOx-treated ARPE-19 cells had no effect with regard to changes in mRNA of inflammatory markers (Fig. 7*D*). Since EVs isolated from the apical site of GOx-treated ARPE-19 cells contained enhanced amounts of mtDNA (Fig. 7*B*), we also directly tested the effects of treatment with isolated mtDNA and nuDNA (1 μg) encapsulated within a lipid bilayer (Lipofectamine 2000) in naïve HMC3 cells. A robust increase in expression of TNFα was detected in HMC3 cells treated only with isolated mtDNA, but not nuDNA (Fig. 7*E*). Finally, we tested which intracellular DNA receptor in microglia binds mtDNA derived from RPE cells. We isolated mtDNA from ARPE-19 cells, amplified it in the presence of bromodeoxyuridine (BrDU), and treated naïve HMC3 cells with BrDU-labeled mtDNA encapsulated within a lipid bilayer (Lipofectamine 2000, Fig. 8*A*). Using the PLA approach, we detected an interaction between BrDU-labeled mtDNA with ZBP1 but not with cGAS, TLR9, AIM2, or NLRP3 (Fig. 8*B*). To further confirm microglia activation by isolated mtDNA, we detected that treatment of HMC3 with isolated mtDNA, but not nuDNA, caused enhanced generation of reactive oxygen species (ROS) in HMC3, as detected by MitoSOX staining (Fig. 8*C*). Together, these data revealed a previously unknown mechanism of intercellular communication by EVs derived from the apical site of RPE cells in activation of microglia. Moreover, our data point toward mtDNA as being a critical signaling molecule in RPE–microglia cross talk and ZBP1 acting as its intracellular receptor.

**Figure 9:**
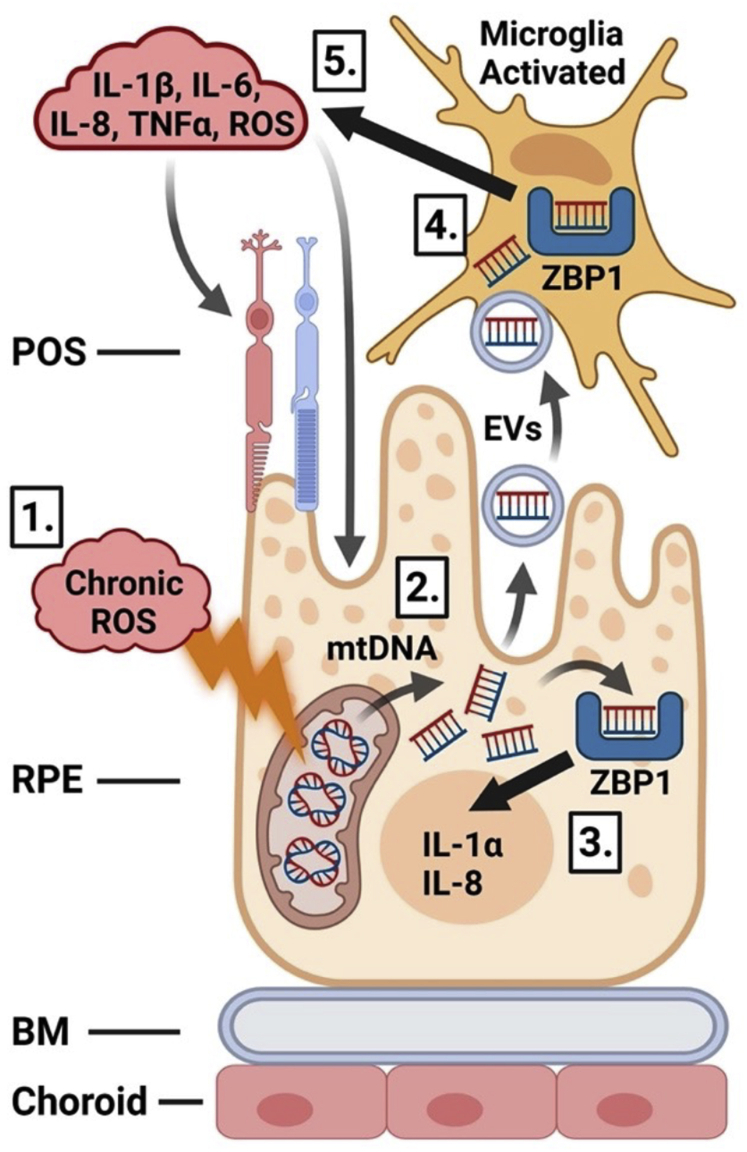
**mtDNA-centered model of development of chronic inflammation in the retina.** Chronic, low-level oxidative stress (*1*) generates a proinflammatory environment in RPE by activation of the mtDNA/ZBP1 pathway (*2* and *3*). Concurrently, RPE cells release EVs containing fragments of mtDNA into the retina that activate the proinflammatory phenotype of microglia (*4*). Chronic activation of mtDNA-mediated RPE–microglia cross talk maintains inflammation in the retina affecting the viability of photoreceptors, ganglion, and RPE cells (*5*). Image created with BioRender.com. BM, Bruch's membrane; POS, photoreceptor outer segment; RPE, retinal pigment epithelial.

In summary, our study shows that chronic oxidative stress that is commonly observed in ocular pathologies results in the induction of mtDNA-specific damage that causes expression of proinflammatory markers in RPE cells *via* activation of ZBP1 signaling. Additionally, polarized RPE cells subjected to oxidative stress release EVs containing mtDNA that are capable of activation of proinflammatory phenotype of microglia. Based on these data, we propose a novel mtDNA-centered mechanism for the generation of a proinflammatory environment in the retina. In our model, low levels of oxidative stress generate a proinflammatory niche by activation of mtDNA/ZBP1 pathway in RPE while releasing EVs containing mtDNA within the retina, which then activates the proinflammatory phenotype of microglia. We suspect that chronic activation of pathological RPE–microglia cross talk affects retina homeostasis resulting in a decreased viability of photoreceptors, ganglion, and RPE cells, among others, contributing to retina dysfunction (Fig. 9). Together, we propose that the molecular mechanisms described in this study contribute to the development of several ocular pathologies and therefore are potentially novel targets for therapeutic interventions.

## Discussion

Our studies identified a central role of oxidatively damaged mtDNA and its intracellular receptor, ZBP1, in activation of microglia and generation of a proinflammatory environment in retina. This mtDNA-centered autocrine and paracrine signaling occurred in response to chronic oxidative stress, a type of insult that is considered to drive several ocular pathologies such as AMD. Additionally, our data highlight that fundamentally different cellular responses are generated by prolonged low levels of enzymatically generated H_2_O_2_ *versus* bolus H_2_O_2_ treatment, suggesting it is critical to consider the importance of the dynamics of oxidatively induced damage. Fully differentiated RPE cells can tolerate exposure to high levels of bolus H_2_O_2_ but are highly susceptible to chronic low doses of H_2_O_2_. We hypothesize that the differential outcomes of bolus *versus* chronic oxidative stress are most likely due to the kinetics of ROS generation. Similar modeling of chronic oxidative stress in analysis of time-developed phenotype has been previously proposed in the ocular field (30). Furthermore, prolonged exposure to low levels of oxidants triggered mtDNA-mediated signaling that is absent in cells treated with bolus amounts of oxidants. Therefore, our report highlights mtDNA as a novel key signaling molecule in response to chronic oxidative stress conditions.

There are several reports detecting greater damage to mtDNA than to nuDNA in retinal cells in aging animals and ocular pathologies. Increased amounts of 8-hydroxy-2′-deoxy-guanosine (8-oxoG), the most frequently occurring DNA lesion, together with increased numbers of DNA breaks and deletions, were reported in mtDNA from the retina of old mice (10). It was proposed that this DNA damage is caused by age-related decrease in the expression of DNA repair enzymes and reduction in DNA repair capacity in aging retinas (10). We previously detected age-related and tissue-specific decrease of DNA repair capacity in mitochondria, along with a decrease in expression of antioxidant enzymes as an underlying cause of age-dependent accumulation of mtDNA damage (31). Increased levels of mtDNA damage in human aged macular, but not the peripheral region of retina, in combination with decreased DNA repair capacity were also reported previously (14). In contrast, another report showed no difference in the amounts of mtDNA damage between the macular and peripheral region in aged human donor eyes, but significant mtDNA damage was found in RPE cells (32). It was shown that the level of mtDNA damage positively correlates with the grading level of AMD (14). An increased amount of mtDNA deletion was suggested to be a result of the aging process, while increased numbers of DNA damage (DNA breaks, DNA lesions) were linked with AMD (13). It was also reported that mtDNA damage accumulates eight times faster than its nuclear counterpart and that in AMD there is an increased enhancement of mtDNA damage (13). All these reports clearly indicate a specific accumulation of mtDNA damage, DNA breaks, and DNA lesions, particularly in RPE cells during aging, a process that is further accelerated in AMD. These reports also strongly support testing the effects of chronic oxidative stress such as that generated by GOx treatment, used in this report, in cultured cells as a model closely reflecting age- and time-related changes.

Numerous reports demonstrate that mtDNA is particularly susceptible to oxidative stress and that oxidative mtDNA damage persists longer than in nuDNA (31, 33, 34, 35, 36, 37, 38, 39, 40). Some of the contributing factors include a reduced number of mtDNA repair pathways (41), enzymes involved in mtDNA repair (42, 43, 44), the close proximity to the mitochondrial respiratory chain, the main intracellular source of ROS (45). Indeed, we found that mtDNA is a primary target for oxidative stress also in fully differentiated ARPE-19 cells. Both bolus and chronic exposure to H_2_O_2_ primarily induce damage to mtDNA, followed by damage to the nuDNA (Figs. 1 and S3*B*). While damage induced by bolus H_2_O_2_ treatment can to some extent be efficiently repaired, GOx-induced time-dependent exposure to oxidants resulted in an increase of mtDNA damage at a rate exceeding mitochondrial DNA repair capacity, causing mitochondrial dysfunction that includes an increase of mitochondrial-derived oxidants, which in turn may further accelerate damage to nuDNA (Fig. S9). Consequently, we detected intracellular translocation of oxidatively damaged mtDNA to the cytoplasm where it stably binds the DNA/RNA receptor ZBP1 (Fig. 2). It was reported that mtDNA liberated from mostly necrotic cells induces inflammatory responses in immune cells by binding to Toll-like receptor 9 (TLR9), cyclic GMP-AMP synthase (cGAS), and nucleotide-binding domain leucine-rich repeat family, pyrin-domain containing 3 (NLRP3) inflammasome (18, 46, 47, 48, 49, 50, 51, 52, 53). ZBP1 was initially identified as an intracellular receptor specific to viral and bacterial DNA and RNA causing activation of type I interferon (28, 54) and NF-kB (28, 54, 55, 56, 57) signaling. Recent reports showed the critical role of ZBP1-mediated signaling in the development of skin (58, 59) and bowel (60) sterile inflammation; however, these reports lacked identification of endogenous nucleic acids that bind and trigger ZBP1 signaling. Our previous report (16) showed for the first time that ZBP1 binds to oxidatively damaged mtDNA and triggers expression of proinflammatory markers in viable lung epithelial cells. Recent reports showed activation of the cGAS-STING pathway by mtDNA liberated from mitochondria and translocated to the cytoplasm in response to pyrimidine deficiency and defective mitophagy, ultimately leads to activation of downstream STING-dependent inflammatory response (61, 62). Our current report shows that exposure of differentiated RPE cells to chronic oxidative stress results in mtDNA damage and its translocation to cytoplasm where it binds and activates ZBP1 without activating cGAS-STING signaling (Figs. 2 and 3). Therefore, an immediate question arises: how does mtDNA released from mitochondria become distinctly recognized by different intracellular DNA/RNA receptors? At this point, we speculate that such differential interactions may be due to the specific type of damage induced by the GOx on mtDNA, or the size of mtDNA fragments released to the cytoplasm; however, these hypotheses need future investigation.

The role of RPE in the development of ocular pathology is extensively studied particularly in connection with AMD. The proper maintenance of mitochondrial homeostasis in RPE cells is crucial since they utilize oxidative phosphorylation for most of their energy production (63). It has been proposed that altered RPE cell metabolism could create a bioenergetic crisis in the retina that drives toward AMD pathology (64). Two independent studies showed reduced oxidative phosphorylation in RPE cells cultured from human donors with AMD when compared with RPE cells isolated from healthy donors (65, 66). Additionally, we have herein detected significant mtDNA damage and remodeling of the major mitochondrial bioenergetic parameters, including a decrease in oxygen consumption linked with ATP production in differentiated ARPE-19 cells subjected to chronic oxidative stress (Figs. 1*C* and S9). Based on our data, we postulate that mitochondrial dysfunction including mtDNA damage may be an active source of retinal dysfunction rather than its passive consequence. We propose that damaged mtDNA serves as an important molecule in autocrine and paracrine-mediated signaling including activation of microglia. It was shown that prolonged microglial activation is detrimental to the cell, causing extensive ROS and inflammatory marker secretion including IL-1β, IL-6, TNFα. The generation of proinflammatory environments fostered by reactive microglia negatively affects retinal homeostasis, leading to cell death of photoreceptors and RPE dysfunction (20). Enlarged, chronically activated amoeboid microglia were found in the retina of AMD subjects (67). Our data showed that RPE-derived EVs containing mtDNA are a potent inducer of microglial activation (Fig. 7), and that persistent, low-level oxidative stress in RPE cells causes a substantial increase in mtDNA containing EV release to the surrounding environment over time (Fig. 5*B*). Thus, we propose that chronic activation of RPE–microglia communication *via* EVs significantly contributes to the generation of a detrimental proinflammatory and pro-oxidative niche in the retina. In fact, reactive microglia induce NLRP3 inflammasome activation in RPE cells that can subsequently induce RPE degeneration *via* caspase-1 mediated pyroptosis (68). ZBP1 is implicated as an upstream regulator of necrotic cell death (69); therefore, we hypothesize that chronic activation of microglia could in turn enhance mtDNA damage in RPE cells causing chronic activation of ZBP1 and subsequent retina degeneration. It is worth noting that while the GOx used in our experiments induces chronic oxidative stress, the amount of generated H_2_O_2_ plateaus by 6 h, most likely due to the decreased enzymatic activity of GOx (Fig. S2). These conditions clearly activated ZBP1-mediated expression of proinflammatory markers without significant decrease of cellular viability. However, we cannot exclude the possibility that constant exposure to oxidants, *e.g.*, by persistently activated microglia, could trigger activation of ZBP1-mediated PANoptosis at later time points.

Finally, our data revealed that fully differentiated, but not replicative RPE cells secrete EVs containing different cargo from the apical and basolateral sites. It was reported that bolus H_2_O_2_ treatment of RPE cells causes secretion of neuroprotective αB-Crystallin from the apical site *via* EVs (70), while stimulation of polarized RPE cells differentially secretes IL-6 and IL-8 from apical and basolateral sites (71). In addition, significantly more EVs were secreted from the apical than the basolateral site of polarized porcine RPE cells (72). We found enhanced amounts of mtDNA present in EVs isolated from differentiated RPE cells that is further enhanced by oxidative stress (Figs. 5 and 7). In addition, we performed initial characterization of the mtDNA derived from RPE cells. Interestingly, although we found a lack of mtDNA sequence specificity, we discovered that large mtDNA fragments are present in EVs released from RPE cells (Fig. 6). Most importantly, our study discovered a previously unknown mechanism of communication mediated by the RPE-derived EVs in cross talk with microglia, which emphasizes the key role of mtDNA. The significance of EVs in cell-to-cell communication, immune modulation, and neovascularization among other functions is well established in cancer, but their role in ocular pathologies is yet to be fully understood (73, 74). In this report we focus on mtDNA, but differential content of EVs released from polarized RPE cells need further investigation since they may contain other important mediators in communication with microglia and other cell types present in the retina. This seems to be particularly important since marked increase of EVs released from GOx-treated cells was measured. Thus, as this point we cannot exclude possibility that mtDNA is only one of the factors present in EVs that activate microglia. We also did note a time-dependent change in the size of EVs released from differentiated RPE cells following GOx exposure (Fig. 5, *C* and *D*), highlighting the dynamic nature of the oxidative stress response and perhaps indicating a shift in cargo content, which has yet to be identified. This observation also needs further analysis.

Altogether, we have described novel underlying molecular mechanisms that may potentially drive the development of chronic inflammation that frequently occurs in ocular pathologies. In our model, chronic low levels of oxidative stress generate a proinflammatory niche in RPE cells by activation of the mtDNA/ZBP1 pathway and subsequent release of EVs containing mtDNA to the apical site, then activation of the proinflammatory phenotype of microglia, which in turn will decrease viability of photoreceptors, ganglion cells, and RPE cells, among others (Fig. 9). Our novel findings highlight various key players in the activation of retinal inflammation and expose new molecular pathways that may be used as target candidates in ocular therapies.

## Experimental procedures

### Cell lines

All cell lines used in this study were obtained from ATCC unless otherwise stated and were maintained in a humidified incubator at 37 °C with 5% CO_2_. Human retinal pigment epithelial cells, ARPE-19 (CRL-2302), were propagated in DMEM:F12 medium (ATCC 30–2006) supplemented with 10% heat-inactivated Fetal Bovine Serum (FBS, GIBCO #10082147) and 100 U/ml of Penicillin-Streptomycin (GIBCO #15140148). ARPE-19 cells were differentiated in DMEM:F12 medium supplemented with 1% heat-inactivated FBS and 100 U/ml of Penicillin-Streptomycin for 3 months with medium changed twice per week. Human brain microglia, HMC3 (CRL-3304), were propagated with EMEM (ATCC 30–2003) supplemented with 10% heat-inactivated FBS (GIBCO #10082147) and 100 U/ml of Penicillin-Streptomycin (GIBCO #15140148). Normal human retinal pigment epithelial cells were obtained from LONZA (#00194987) and were propagated using retinal pigment epithelial growth media (#00195409, #001954406, #00195407). In this study we used FBS that was depleted from EVs by ultracentrifugation for 18 h at 100,000*g*. Mouse embryonic fibroblasts (MEFs) derived from WT and ZBP1 KO C57BL/6J mice were isolated from fetuses at embryonic day 13.5/14.5 in accordance with a standard protocol (75).

### Glucose oxidase (GOx) and hydrogen peroxide (H_2_O_2_) treatment

To generate constant, low-level oxidative stress, cells were treated with various concentration of glucose oxidase, GOx (SIGMA #G2133–50KU). GOx was resuspended in phosphate buffered saline (PBS) to prepare 100X stock that was diluted into to the culture medium. To generate bolus oxidative stress, cells were treated with various concentrations of H_2_O_2_ (EMD Millipore #HX0635–3).

### Western blotting analysis

Cells (∼300,000) were lysed using NP-40 buffer (150 mM NaCl, 50 mM Tris-Cl pH8.0, 1% NP-40) for 30 min on ice followed by brief centrifugation to separate the insoluble fraction. Total protein concentration was determined using DC Protein Assay (BioRad #5000111) according to manufacturer's recommendation, and 40 μg of protein was precipitated with four volumes of acetone overnight, followed by centrifugation at 21,000*g* for 15 min at 4 °C. The pellet was washed with 70% EtOH followed by centrifugation at 21,000*g* for 5 min at 4 °C. Finally, the pellet was resuspended in 2X Laemmli Sample Buffer (BioRad #161–0737), incubated at 95 °C for 2 min and loaded on NuPAGE 4 to 12% Bis-Tris acrylamide gel (Invitrogen #NP0322). Proteins were transferred to Trans-Blot Transfer Medium Pure Nitrocellulose (BioRad #162–0146). Membranes were blocked with 5% nonfat milk in TBS (20 mM Tris pH 7.4, 136 mM NaCl) containing 0.05% Tween-20 and subsequently probed overnight at 4 °C (for primary) and for 1 h at room temperature (for secondary) antibodies: MCT3 (abcam #60333), CRALBP (abcam #15051), caspase-3 (Cell Signaling #9665S), HRP-linked anti-mouse (Cell Signaling #7076S), HRP-linked anti rabbit (Cell Signaling #7074S), HRP-linked β-actin (Cell Signaling #1262P). Amersham ECL Western Blotting Detection Reagents (#RPN2106) and G:Box (Syngene) with GeneSnap software were used for visualization and quantification of the signal.

### MTT assay

To measure changes in the metabolic activity of differentiated ARPE-19 cells, 30,000 cells were seeded per well in a 96-well plate and differentiated in a medium containing 1% FBS for 3 months. Next, a solution containing 1:10 of culture medium to MTT reagent (3 mg/ml, Millipore #475989) was incubated in a humidified incubator at 37 °C with 5% CO_2_ for 1 h. Culture medium was removed, and cells were resuspended in DMSO (Fisher #D128). The absorption was measured at 590 nm using a SpectraMax (Molecular Devices) microplate reader with SoftMax Pro 5.3 software. Four technical replicates were measured for each readout.

### LDH assay

Necrotic cell death was assayed by the quantification of activity of lactate dehydrogenase (LDH) release to the culture medium. Briefly, 30,000 cells were seeded per well in a 96-well plate and differentiated in a medium containing 1% FBS for 3 months. Fifty microliters of culture medium was mixed with 50 μl of 200 mM Tris pH 8.0 (SIGMA #T3253), 50 μl of 50 mM Lithium Lactate (SIGMA #L1500), and 50 μl of NAD/PMS/INT solution (SIGMA #N0632, #P9625, #I8377) followed by absorbance reading at 490 nm over 15 min using the SpectraMax (Molecular Devices) microplate reader with SoftMax Pro 5.3 software. Four technical replicates were measured for each readout.

### Mitochondrial and nuclear DNA damage

To measure changes in DNA integrity (damage), we conducted a gene-specific semiquantitative PCR assay (76). Briefly, 300,000 of ARPE-19 cells were seeded per well in 24-well plates and differentiated in medium containing 1% FBS for 3 months. Total DNA was isolated from control and treated cells using DNeasy Blood & Tissue Kit (QIAGEN #69506) according to manufacturer's recommendations. Integrity of the mitochondrial DNA (mtDNA) was assessed using two pairs of primers with amplicons of 211 bp and 8.9 kb for human mtDNA and 117 bp and 10 kb for mouse mtDNA. The short mtDNA amplicon was used as a normalization factor of mtDNA copies (78). Nuclear DNA (nuDNA) integrity was assessed using a 10 kb amplicon. *Taq* DNA Polymerase and LongAmp *Taq* DNA polymerase (NewEngland BioLabs #M0273X and #M0323L) were used for amplification of short and long DNA targets, respectively. DNA integrity in untreated (control) cells was set as 100%. The following primers were used: h-mt221bp FW 5′-CCCCACAAACCCCATTA CTAAACCCA-3′, RV 5′-TTTCATCATGCGGAGATGTTGG ATGG-3′; h-mt8.9kb FW 5′-TCTAAGCCTCCTTATTCGAG CCGA-3′, RV 5′-TTTCATCATGCGGAGATGTTGGATGG-3′; h-nuDNA10kb FW 5′-TGGGATTACACGTGTGAACCA ACC-3′, RV 5′-GCTCTACCCTGTCCTCTACCGTCC-3′; m-mt117bp FV 5′-CCCAGCTACTACCATCATTCAAGT-3′, RV 5′-GATGGTTTGGGAGATTGGTTGATGT-3′; m-mt10kb FV 5′-GCCAGCCTGACCCATAGCCATAATAT-3′, RV 5′-GAGAGATTTTATGGGTGTAATGCGG-3′, m-nuDNA10kb FV 5′-TATCTCTCTTCCTCTTCACTTCTCCCCTGG-3′, RV 5′-CGTGATGCCGCCGTTGAGGGTCTCCTG-3′. We used three technical replicates for each PCR reaction.

### Proximity ligation assay (PLA)

ARPE-19 cells were seeded at a density of 20,000 cells/well in Lab-Tek Chamber Slides w/Cover (Thermo Scientific) and differentiated in medium containing 1% FBS for 3 months. HMC3 were seeded at a density of 20,000 cells/well in Lab-Tek Chamber Slides w/Cover (Thermo Scientific) followed by treatment with bromodeoxyuridine (BrDU) labeled mtDNA isolated from ARPE-19 cells. Cells were fixed and permeabilized with 4% paraformaldehyde, 0.2% Tween-20 in PBS from 20 min. After three washes with PBS, PLA was performed according to manufacturer's instructions using the Duolink *In Situ* Kit (SIGMA #DUO92002-100RXN) with the following pairs of primary antibodies: dsDNA (abcam #27156), TFAM (rGeneTex #GTX44824), ZBP1 (Protein Tech #13285-1-AP), TLR9 (Cell Signaling #5845S) NLRP3 (Cell Signaling #13158S), cGAS (Cell Signaling #15102S), RIP1 (BD Pharmingen #551041), RIP3 (abcam #209384), STING (Cell Signaling #13647), IRF3 (abcam#124399), TBK1 (Thermo Scientific #108A429), BrDU (Sigma #B8434). Images were captured using a Nikon eclipse 80i inverted fluorescent microscope with Photometric CoolSNAP HQ2 camera and NIS-Elements BR 3.10 software. The interaction between proteins was visualized using a red fluorescent signal. In colocalization experiments, mitochondria were probed with rabbit polyclonal antibody against subunit B of ATPase antibody (ATPB, abcam #128743) for 1 h followed by goat anti-mouse AlexaFluor 488 (Thermo Fisher Scientific #A28175) prior to PLA.

### RNA extraction and RT-qPCR

Total RNA was extracted from 300,000 MEFs, HMC3, or differentiated ARPE-19 cells using the RNeasy Mini Kit (QIAGEN #74104) and reverse transcribed with the High-Capacity cDNA Reverse Transcription Kit (Applied Biosystems #4368814) according to the manufacturer's recommendation. The cDNA obtained from 1 to 2 μg of RNA was used for qPCR analysis using the Maxima SYBR Green/ROX qPCR Master Mix (Thermo Scientific #K0221) and CFX96 TouchTM Real-Time PCR Detection System (Bio-Rad) with the following primers: h-IL-1β FW 5′-AGTAGCAACCAACGGGAAGG-3′, RV 5′-CTTCCTCTGAGTCATTGGC GA-3′; h-IL-6 FV 5′-AGTGAGGAACAAGCCAGAGC-3′, RV 5′-ATTTGTGGTTGGGTCAGGGG-3′; h-IL-8 FV 5′-TGTAC TCATGACCAGAAAGACC-3′, RV 5′-GGACACTACTGGG AGTGACAA-3′; h-TNFα FV 5′-GATTCTGAGCAAAATAG CCAGCA-3′, RV 5′-GGCTTCCTTCTTGTTGTGTGT-3′; h-VEGF FV 5′-CTACCTCCACCATGCCAAGT-3′, RV 5′-GCA GTAGCTGCGCTGATAGA-3′; h-IL-4 FV 5′-CAACTGCT TCCCCCTCTGTT-3′, RV 5′- TCTGCTCTGTGAGGCTGT TC-3′; m-IL1α FW 5′-GTCGGGAGGAGACGACTCTA-3′, RV 5′-GCAACTCCTTCAGCAACACG-3′; m-IL-6 FV 5′-GG AGTCACAGAAGGAGTGGC-3′, RV 5′-AGGTTTGCCGAG TAGATCTCAA-3′; m-KC 5′-AGACCATGGCTGGGATTCAC-3′, RV 5′-CGCGACCATTCTTGAGTGTG-3′; m-ZBP1 FV 5′-CCCCTGCGATTATTTGTCAGC-3′, RV 5′-TGACCAATCTGGATGGCGTT-3′; using the following cycle: 95 °C for 10 min, 40 cycles at 95 °C for 15 s, and 60 °C for 1 min. The expression of the interleukins was normalized to *ACTB*. We used ddCt method for qRT-PCR data analysis. We used two technical replicates for each qPCR reaction.

### ZBP1 immunoprecipitation (IP)

Differentiated ARPE-19 cells (1 million cells) were transfected with 100 ng of pCAGGS-HA-hZBP1 or empty expression vector (generous gift from Dr Taniguchi, University of Tokyo, Japan) using Lipofectamine 2000 (Thermo Fisher Scientific, #11668019). After 48 h, cells were washed once with PBS and treated with PBS (control) or GOx (30 mU/ml) for 1 h. Cells were washed with PBS, and DNA–protein cross link was performed by addition of formaldehyde (0.75%) for 10 min, followed by addition of glycine (125 mM) for 10 min. Cells were washed twice with PBS and lysed using NP-40 buffer (150 mM NaCl, 50 mM Tris-Cl pH8.0, 1% NP-40) for 30 min on ice followed by brief centrifugation to separate the insoluble fraction. Protein concentration was determined using DC Protein Assay (BioRad #5000111) according to the manufacturer's recommendation. In total, 200 μg of total cell extract was used for IP using Pierce Magnetic HA-Tag IP/co-IP Kit (Thermo Scientific, #88838) according to manufacturer's recommendations. Total DNA from IPs was isolated using DNeasy Blood & Tissue Kit (QIAGEN #69506) according to manufacturer's recommendations. The amounts of mtDNA and nuDNA were analyzed by qPCR with CFX96 TouchTM Real-Time PCR Detection System (Bio-Rad) and Maxima SYBR Green/ROX qPCR Master Mix (Thermo Scientific #K0221) with the following primers: mtDNA (located in *COXIII* gene): FW 5′-TGACCCACCAATCACATGC-3′, RV 5′-ATCACATGGCTAGGCCGGAG-3′; nuDNA (located in *ACTB* gene): FW 5′-CATGTACGTTGCTATCCAGGC-3′, RV 5′-CTCCTTAATGTCACGCACGAT-3′. We used the following thermal cycle: 95 °C for 10 min, 40 cycles at 95 °C for 15 s, and 60 °C for 1 min.

### Cellular bioenergetics

Analysis of mitochondrial respiration was assessed using the XF24 Extracellular Flux Analyzer (Agilent). Briefly, ARPE-19 cells were seeded on cell culture microplates (60,000/well) and differentiated for 3 months using 1% FBS-containing medium. For analysis of mitochondrial respiration, cells were washed twice with low-glucose DMEM medium pH 7.4 supplemented with L-Glutamine (2 mM, GIBCO #25030149) and sodium pyruvate (0.33 mM, SIGMA #S8636). After 1 h incubation at 37 °C in CO_2_-free incubator, the oxygen consumption rate (OCR) after treatment with oligomycin (1.5 μg/ml, SIGMA #O4876) was used to assess ATP production rate and the OCR after treatment with carbonyl cyanide-4-trifluoromethoxy phenylhydrazone (FCCP, 0.5 μM, SIGMA #C2920) to assess maximal mitochondrial respiratory capacity. Antimycin A (2 μg/ml, SIGMA #A8674) and rotenone (2 μM, SIGMA #R8875) were used to inhibit the ﬂux of electrons through complex III and I, to detect residual nonmitochondrial OCR, which is considered to be due to cytosolic oxidase enzymes. We used four technical replicates for each readout.

### Extracellular vesicle (EVs) isolation and characterization with nanoparticle tracking analysis (NTA)

We isolated EVs derived from 300,000 differentiated ARPE-19 cells. EVs released to the culture medium were isolated using the ultracentrifugation approach as described in (77). This method is commonly used to isolate “small EVs” including exosomes and microvesicles. Briefly, to isolate exosomes/microvesicles, we performed three subsequent centrifugation cycles to separate cellular debris from EVs: 2000*g* 10 min, 20,000*g* 20 min and 100,000*g* for 2 h, all at 4 °C. The pellet of EVs obtained was resuspended in PBS for subsequent assays. The number and size of EVs were analyzed with NTA using NanoSight NS300 (Malvern Panalytical). Briefly, 1 ml of conditioned media samples was collected followed by differential centrifugation (1000*g* 5 min, 5000*g* 10 min, 10,000*g* 30 min, at 4 °C) to clear cell debris and large vesicles before analysis or ultracentrifugation (100,000*g* at 4 °C for 2 h). In some experiments, post ultracentrifugation supernatant was carefully removed and placed in a separate tube while the EV pellet was resuspended in 0.22 μm-filtered PBS to maintain the initial concentration. Samples were then recorded by NTA (3 capture intervals of 60 s per sample with camera level 7, threshold 2). To rule out sedimentation of protein aggregates, in control experiments, we treated EVs with active or inactive Proteinase K (0.1 mg/ml) for 30 min at 37 °C before NTA. We inactivated Proteinase K by incubation at 95 °C for 15 min. Three technical replicates were used for each readout.

### Quantitative real-time PCR (qPCR)

To quantify mtDNA and nuDNA in EVs released from 300,000 differentiated ARPE-19 cell, we isolated total DNA using DNeasy Blood & Tissue Kit (QIAGEN #69506) according to manufacturer's recommendations. We used the CFX96 TouchTM Real-Time PCR Detection System (Bio-Rad) and Maxima SYBR Green/ROX qPCR Master Mix (Thermo Scientific #K0221) with the following primers: mtDNA (located in *COXIII*gene): FW 5′-TGACCCACCAATCACATGC-3′, RV 5′-ATCACATGGCTAGGCCGGAG-3′; nuDNA (located in *ACTB* gene): FW 5′-CATGTACGTTGCTATCCAGGC-3′, RV 5′-CTCCTTAATGTCACGCACGAT-3′. We used the following thermal cycle: 95 °C for 10 min, 40 cycles at 95 °C for 15 s, and 60 °C for 1 min. To determine mtDNA content in differentiated ARPE-19 cells, the expression of mtDNA-specific genes (*COXIII*) was compared with the expression of a nuclear gene (*ACTB*) that was used as a control/normalizing factor. For testing mtDNA encoded genes in mtDNA present in EVs, we used following primers: 16S FW 5′-AACTTTGCAAGGAGAGCCAAAGC-3′, RV 5′-GGGATTAGAGGGT TCTGTGGGC-3′; ND1 FW 5′-ACGCCATAAAACTCTTC ACCAAAG, RV 5′-TAGTAGAAGAGCGATGGTGAGAGCT A-3′; ND3 FW 5′-CATTTTGACTACCACAACTCAACGGC TAC-3′, RV 5′-GGGTAAAAGGAGGGCAATTTCTAGATC-3′; ND4 FW 5′-CCAACGCCACTTATCCAGTG-3′, RW 5′-G GGAAGGGAGCCTACTAGGGTGT-3′; ND4L FW 5′-CCAA CGCCACTTATCCAGTG-3′, RV 5′-AGGCCATATGTGTTG GAGATTGAGA-3′, ND5 FW 5′-TTACCACCCTCGTTAAC CCTAACAAA-3′, RV 5′-TGGGTTGTTTGGGTTGTGGCT-3′; ND6 FW 5′-ACGCCCATAATCATACAAAGCCC-3′, RW 5′-GGATTGGTGCTGTGGGTGAAA-3′; Cytb FW 5′-CGCC TGCCTGATCCTCCAA-3′, RV 5′-AGGCCTCGCCCGATGT GTAG-3′; COXI FW 5′-TGCCATAACCCAATACCAAACG C-3′, RV 5′-CTGTTAGTAGTATAGTGATGCCAGCAGCT AGG-3′; COXII FW 5′-CTACGGTCAATGCTCTGAAATCT GTG-3′, RV 5′-GCTAAGTTAGCTTTACAGTGGGCTCTA G-3′; COXII FW 5′-CGATACGGGATAATCCTATTTATTA CCTCAG-3′, RW 5′-CAGGTGATTGATACTCCTGATGCG A-3′; D-loop FW 5′-TGGCCACAGCACTTAAACACATCT C-3′, RV 5′-GGGTTGTATTGATGAGATTAGTAGTATGG GAG-3′. The amplicon size for these primers varies between 150 and 230 bp. Each qPCR reaction was run technical duplicates.

### Enzyme-linked immunosorbent assay (ELISA)

The amount of TNFα in cultured medium of HMC3 microglia was determined using human TNF ELISA Set (BD-Bioscience #555212) according to the manufacturer's recommendation. In total, 300,000 HMC3 cells were seeded per well in 12-well plates and treated with mtDNA or nuDNA for 24 h using Lipofectamine 2000 (Thermo Fisher Scientific, #11668019), according to manufacturer's recommendations. The amount of IL-8 in medium and isolated EVs derived from 300,000 differentiated ARPE-19 cell were determined using human IL-8/CXCL8 DuoSet ELISA (R@D System, #DY208–05).

### MitoSOX red staining

A mitochondrial superoxide indicator was used to detect mitochondrial ROS production. Briefly, HMC3 (40,000 cells/well) were seeded in Lab-Tek II chamber slides (Nalge Nunc International, #154526PK) and incubated at 37 °C, 5% CO_2_ humidified incubator overnight. The cells were treated with 100 ng of isolated from ARPE-19 cell mtDNA or nuDNA encapsulated within Lipofectamine 2000 (Thermo Fisher Scientific, #11668019), for 1 h followed by addition of 5 μM of MitoSOX Red dye (Invitrogen #M36008) for 10 min. For analysis, cells were washed three times with PBS, and the dye's specific fluorescence was visualized using a Nikon eclipse 80i inverted microscope with Photometric CoolSNAP HQ2 camera and NIS-Elements BR 3.10 software.

### Analysis of mtDNA in EVs

Total DNA was isolated from EVs sedimented from 300,000 differentiated ARPE-19 cells. PCR primers were designed using the NCBI Primer-BLAST tool with reference mtDNA (accession number NC_012920.1). For each PCR reaction (25 μl), 2 μl of sample DNA and 0.5 μl of each primer (10 mM) were used together with other components according to manufacturer's recommendations for Standard *Taq* and Long Amp *Taq* DNA Polymerases (both from New England BioLabs, #M0273S, M0323S). Using a Thermocycler C1000 Touch (BioRad), the PCR programs include initial denaturing step at 95 °C for 3 min followed by 32 cycles of 95 °C for 30 s, 55 °C for 30 s, and 68 °C for 1 min, and final extension at 68 °C for 5 min. We used 1 min extension step for amplicons 200 bp-1 kb and 6 min for amplicons 3–6 kb. The PCR products were separated using 1% agarose gel and visualized using G:Box with GeneSnap software. We used following primers: site I FW1 5′-TCAACCTCACCACCTCTTGC-3′, RV1.1 5′-GGCC CTGTTCAACTAAGCAC-3′, RV1.2 5′-TTGCGCCAGGTTT CAATTTCT-3′, RV1.3 5′-GTGGGTGTTGAGCTTGAACG-3′, RV1.4 5′-CAGGGAGGTTAGAAGTAGGGTC-3′, RV1.5 5′-CCGGATAGGCCGAGAAAGTG-3′. Site II: FW2 5′-ACG TTGTAGCCCACTTCCAC-3′, RV2.1 5′-GGGGTAGTCCGAGTAACGTC-3′, RV2.2 5′-CGCTGCATGTGCCATTAAGATA-3′, RV2.3 5′-TCAACGTCAAGGAGTCGCAG-3′, RV2.4 5′-ACAAAATGCCAGTATCAGGCG-3′, RV2.5 5′-GG TGAGGCTTGGATTAGCGT-3′. Site III: FW3 5′-ACGCTAATCCAAGCCTCACC-3′, RV3.1 5′-GTGGTGATAGCGC CTAAGCA-3′, RV3.2 5′-AATGCTAGGCTGCCAATGGT-3′, RV3.3 5′-TTGTGCGGTGTGTGATGCTA-3′, RV3.4 5′-GAG GATGGTGGTCAAGGGAC-3′.

### Statistical analysis

All statistical analyses were performed using GraphPad Prism software. Except where otherwise stated, presented data are based on at least three biological replicates (cells at different passage number or differentiated while in different passage number; n ≥ 3). In addition, most of the techniques have their technical replicates as indicated above. After performing a normality test of obtained data, we used *t*-tests to determine statistical significance between two groups. In the analysis of more than two groups, after performing normality tests, a One-Way ANOVA Kruskal–Wallis test was used. Significant differences are denoted as: ∗*p* < 0.05, ∗∗ *p* < 0.01.

## Data availability

All data supporting the results presented herein are available from the article paper.

## Supporting information

This article contains supporting information.

## Conflict of interest

The authors declare no conflict of interest in regard to this manuscript.
