# Supplementary material for: Oxidative stress induces Z-DNA-binding protein 1–dependent activation of microglia via mtDNA released from retinal pigment epithelial cells
Source: J Biol Chem. 2021 Dec 23;298(1):101523. doi: 10.1016/j.jbc.2021.101523 (PMC8753185; doi:10.1016/j.jbc.2021.101523)
Supplement: Supporting information [file mmc1.docx]

**Supporting information**

Oxidative stress induces Z-DNA binding protein 1-dependent activation of microglia via mtDNA released from retinal pigment epithelial cells.

Jamal Saada^1,2,#^, Ryan J. McAuley^3#^, Michela Marcatti^2,4^, Tony Zifeng Tang^3^, Massoud Motamedi^1^, and Bartosz Szczesny^1,2,*^

Departments of ^1^Ophthalmology and Visual Sciences, ^2^Anesthesiology, ^3^Neuroscience, Cell Biology and Anatomy, ^4^Neurology, University of Texas Medical Branch, Galveston, TX, USA;

# equal contribution

^*^Corresponding author:

Bartosz Szczesny PhD

University of Texas Medical Branch at Galveston

Research Building 21, 4.202J

301 University Boulevard

Galveston, TX 77555-1156

Phone: (409) 747-5383

e-mail: baszczes@utmb.edu (B.S.)

**Supporting methods**

**Quantification of H_2_O_2_ generated by glucose oxidase.** To measure the amount of H_2_O_2_ generated by glucose oxidase (GOx) we used Amplex Red Hydrogen Peroxide/Peroxidase Assay Kit (Invitrogen, #A22188). We used various concentrations of stabilized H_2_O_2_ solution as a reference. We used 10 mU of GOx per ml of medium and the amount of generated H_2_O_2_ was measured at 10 min, 0.5h, 1h, 3h, 6h and 24h according to the manufacturer's recommendation.

**Supporting figures and figure legends**

**
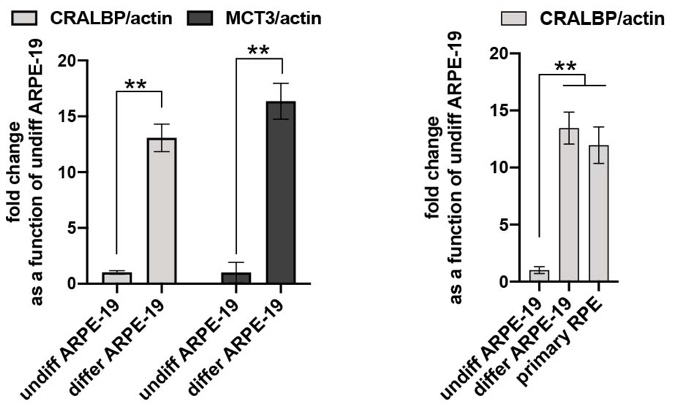
**

**Figure S1.** **Differentiated ARPE-19 cells regain characteristics of primary RPE cells**. Analysis of CRLBP and MCT3 expression in undifferentiated and differentiated ARPE-19 and primary RPE cells shown as fold changes relative to undifferentiated ARPE-19. ** p<0.01 vs undifferentiated ARPE-19 based on t-test analysis. Data is based on n=3 and is expressed as mean ±SD.


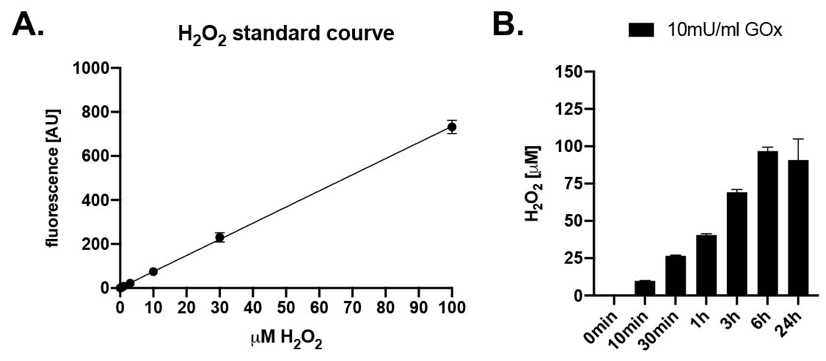


**Figure S2. Quantification of the amount of H_2_O_2_ generated by glucose oxidase.** Standard curve of several H_2_O_2_ concentrations measured by Amplex Red (left graph). The amount of H_2_O_2_ generated by 10 mU/ml of GOx over time. Note the linear increase of H_2_O_2_ generation by GOx up to 6h. 10 mU/ml of GOx generates an equivalent of 100 mM H_2_O_2_ within 6h. Data expressed as mean ± SD is based on n=2.

**
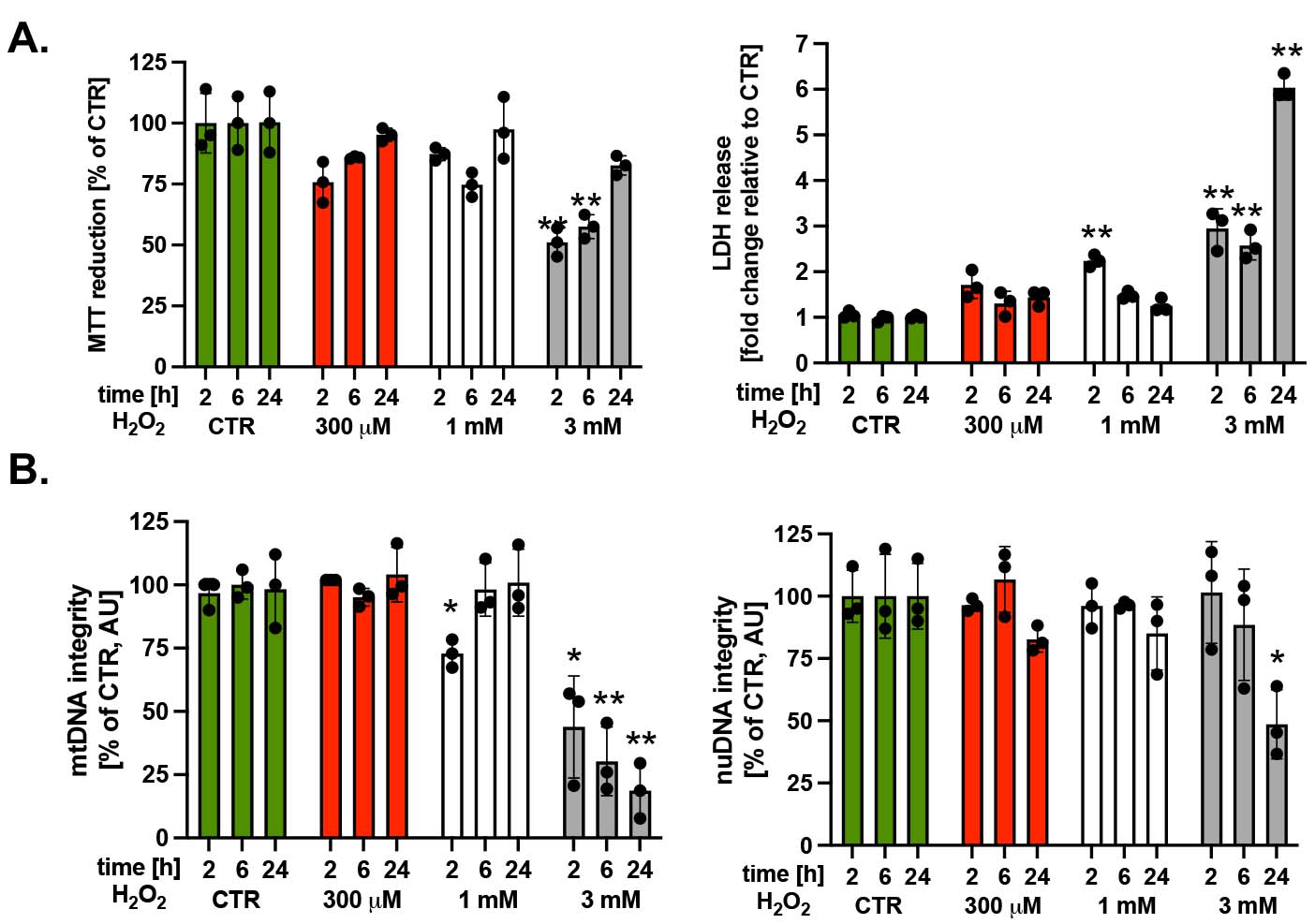
**

**Figure S3. Metabolic status, necrotic cell death and DNA damage of differentiated ARPE-19 exposed to H_2_O_2_.** Time- and concentration-dependent changes in the (A) metabolic status (MTT assay, left panel) and necrotic cell death (LDH release, right panel) and (B) mtDNA damage (left panel) and nuDNA damage (right panel) measured with LA-qPCR of ARPE-19 cells treated H_2_O_2_. Data is based on n=3 and is expressed as mean ± SD. *p<0.05, **p<0.01 vs control (untreated) cells based on one-way ANOVA multiple comparison tests.


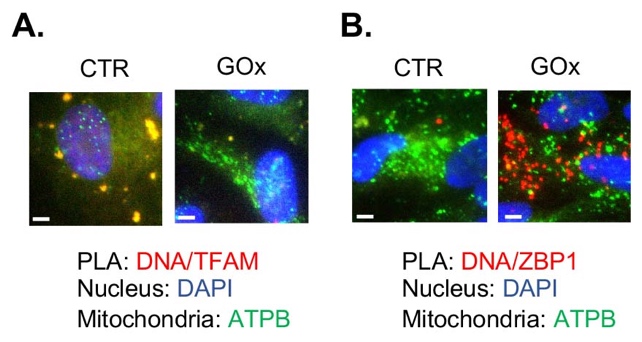


**Figure S4. mtDNA/ZBP1 interaction occurs in cytoplasm.** (A) Co-localization between DNA/TFAM PLA with mitochondrial marker, ATPB, occurs only in control but not in GOx-treated differentiated ARPE-19 cells. (B) Lack of co-localization between DNA/ZBP1 PLA with the mitochondrial marker, ATPB, in control and GOx-treated differentiated ARPE-19 cells. Data indicate mitochondrial and cytoplasmic localization of DNA/TFAM and DNA/ZBP1, respectively. Scale bar, 10 μm.

**
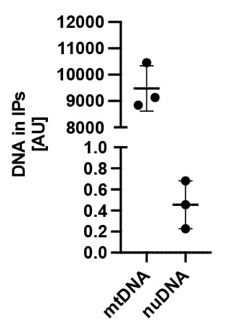
**

**Figure S5. Marked difference between mtDNA and nuDNA in ZBP1-HA IPs.** The amount of mtDNA and nuDNA in ZBP1-HA IPs was quantified using qPCR. mtCOXIII- and nuACTB-specific primers were used. Data is based on n=3 and is expressed as mean ± SD.


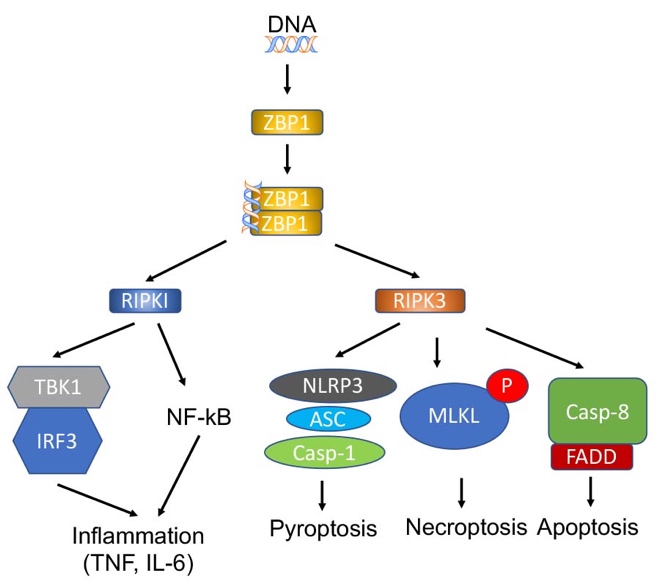


**Figure S6. Schematic representation of DNA-activated ZBP1 signaling pathways.**


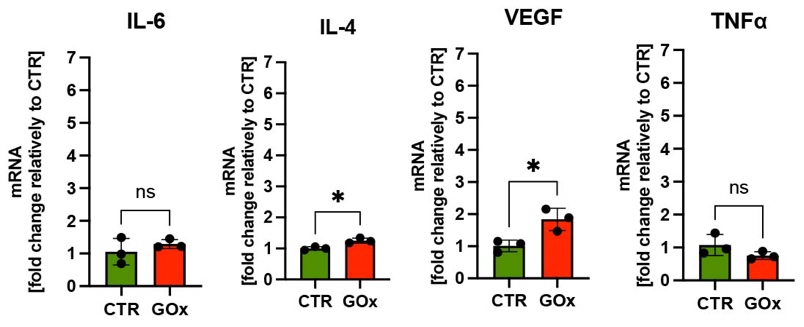


**Figure S7. Effect of chronic oxidative stress on expression of pro-inflammatory markers.** Changes in expression of IL-6, IL-4, VEGF and TNFα in control and GOx-treated (30 mU/ml) differentiated ARPE-19 cells at 24h. Data is based on n=3 and expressed as mean ± SD. *p<0.05 vs control (untreated) cells based on t-tests.


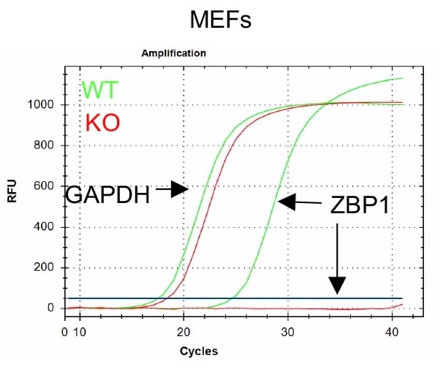


**Figure S8. Lack of Z-DNA binding protein 1 expression in mouse embryonic fibroblasts isolated from ZBP1 KO mice in comparison to WT C67Bl/6J mice.** The lack of ZBP1 expression was analyzed with RT-qPCR. GAPDH was used as an internal control. Note the lack of difference in MEFS derived from WT and ZBP1 KO mice in the amplification curve for GAPDH versus the lack of amplification for ZBP1 in KO MEFs, confirming depletion of ZBP1.


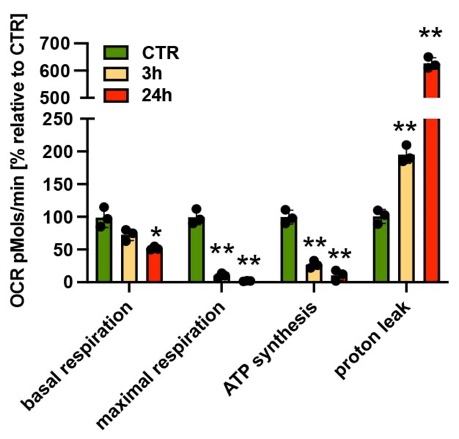


**Figure S9. Chronic oxidative stress causes mitochondrial dysfunction in differentiated ARPE-19 cells.** Time-dependent decrease of oxygen consumption linked with all major bioenergetics parameters and increased proton leakage in ARPE-19 cells treated with 30 mU/ml of GOx. Changes in oxygen consumption were analyzed using Extracellular Flux Analyzer and expressed relative to untreated controls. Data is based on n=3 and is expressed as mean ± SD. *p<0.05, **p<0.01 vs control (untreated) cells based on one-way ANOVA multiple comparison tests.

**
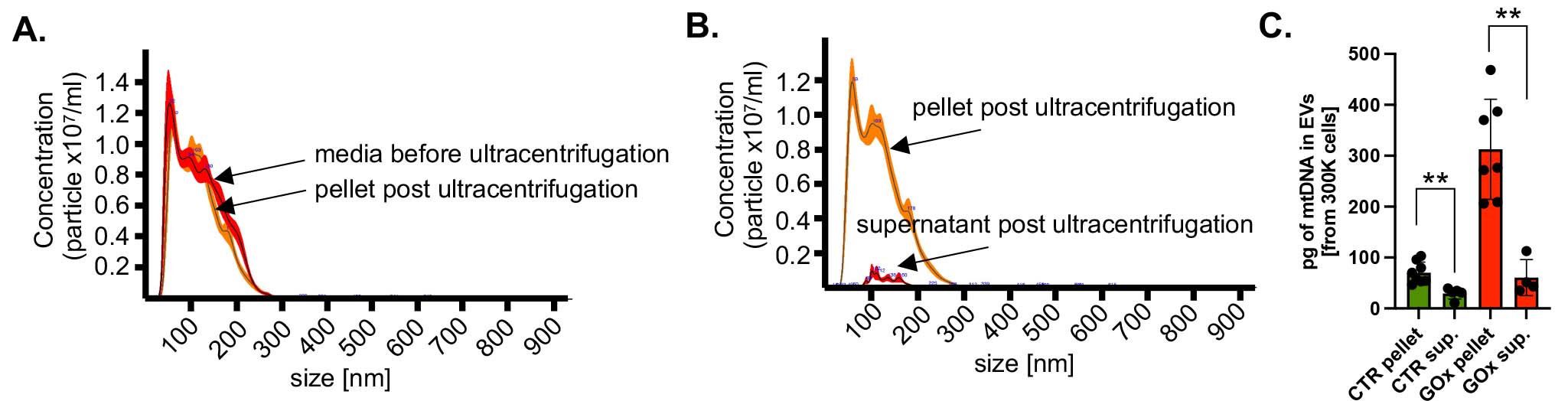
**

**Figure S10. Characterization of EVs released from differentiated ARPE-19 cells at 24h post GOx-treatment.** (A) The same size profile of EVs is present in pre-cleared medium and post ultracentrifugation. (B) The majority of EVs present in medium are sedimented by ultracentrifugation. (C) Most of the mtDNA released from ARPE-19 cells are localized in EVs. Combined profiles of n=3 are shown (A,B). Data is based on n=7 and is expressed as mean ± SD. **p<0.01 vs control/GOx pellet based on t-test (C).

**
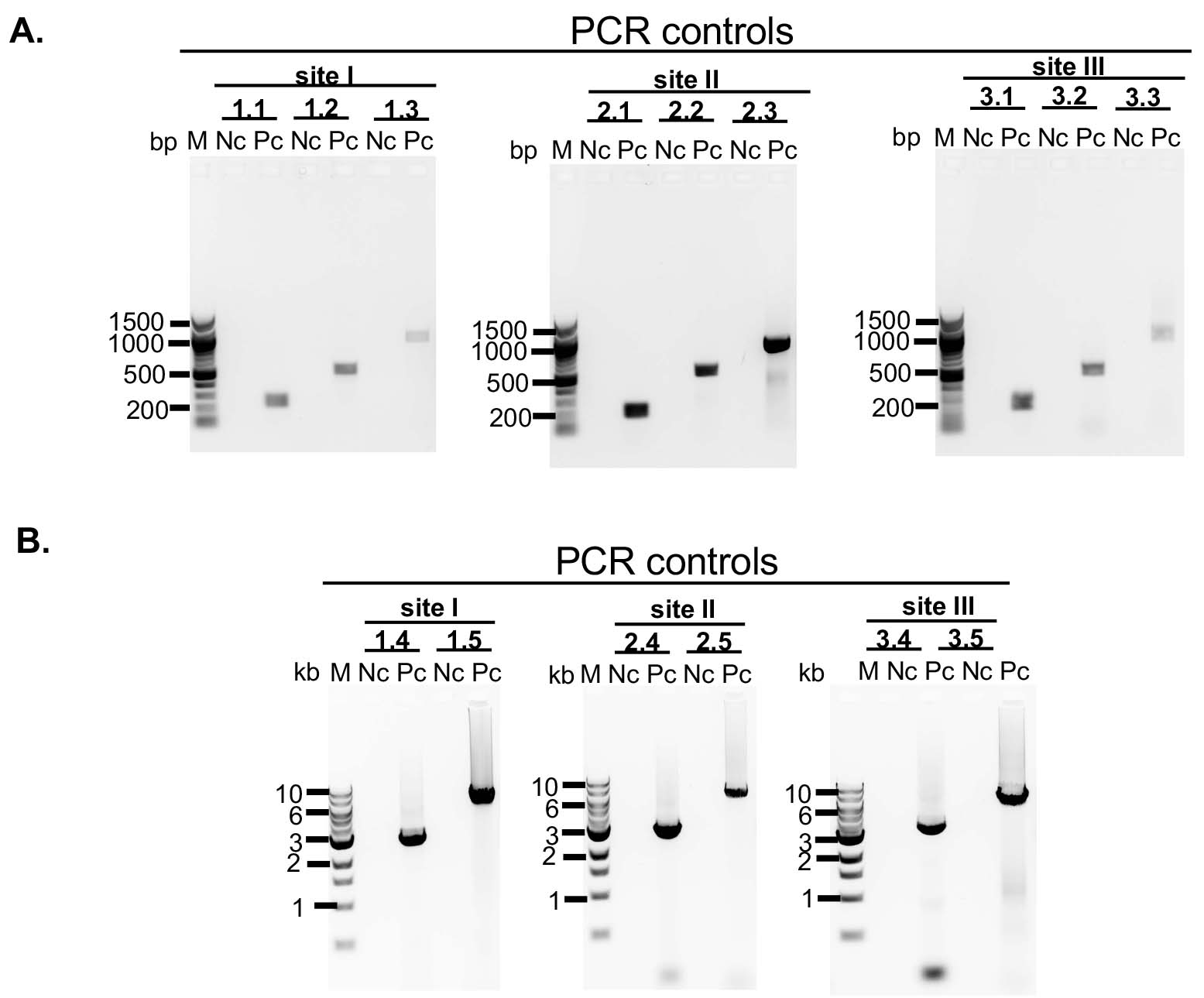
**

**Figure S11. PCR primer controls for analysis of mtDNA in EVs derived from differentiated ARPE-19 cells.** Total DNA isolated from differentiated ARPE-19 cells were used.


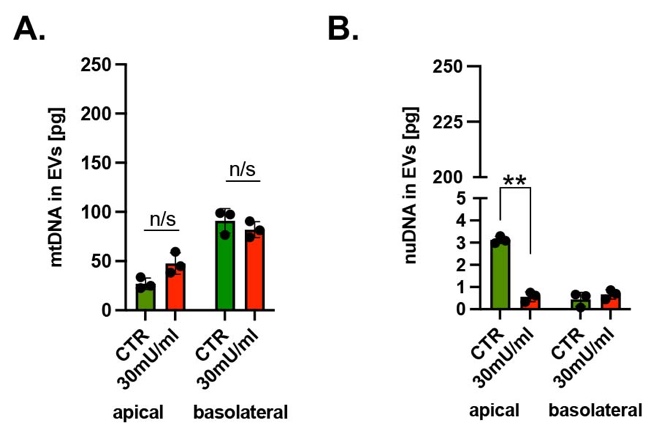


**Figure S12. Chronic, low-levels of oxidative stress do not stimulate release of DNA-containing EVs from GOx-treated replicating, undifferentiated ARPE-19 cells.** Quantification of (A) mtDNA and (B) nuDNA in EVs isolated from apical and basolateral sites of control and undifferentiated ARPE-19 cells treated with 30 mU/ml of GOx at 24h post-treatment. Note, no significant changes in the amount of mtDNA in EVs released from apical and basolateral sites of undifferentiated ARPE-19 cells were found post GOx treatment. Data is based on n=3 and is expressed as mean ± SD. *p<0.05, **p<0.01 vs control/apical site based on t-tests.
